# Enzyme repurposing of a hydrolase as an emergent peroxidase upon metal binding†
†Electronic supplementary information (ESI) available: Experimental details, additional characterization and catalytic data. See DOI: 10.1039/c5sc01065a


**DOI:** 10.1039/c5sc01065a

**Published:** 2015-05-07

**Authors:** Nobutaka Fujieda, Jonas Schätti, Edward Stuttfeld, Kei Ohkubo, Timm Maier, Shunichi Fukuzumi, Thomas R. Ward

**Affiliations:** a Department of Chemistry , University of Basel , Spitalstrasse 51 , CH-4056 Basel , Switzerland . Email: fujieda@mls.eng.osaka-u.ac.jp ; Email: thomas.ward@unibas.ch; b Biozentrum , University of Basel , Klingelbergstr. 50/70 , CH-4056 Basel , Switzerland; c Department of Material and Life Science , Graduate School of Engineering , Osaka University , ALCA and SENTAN , Japan Science and Technology Agency (JST) , 2-1 Yamada-oka , Suita , Osaka 565-0871 , Japan; d Department of Bioinspired Science , Ewha Womans University , Seoul 120–750 , Korea; e Faculty of Science and Technology , Meijo University and ALCA and SENTAN , Japan Science and Technology Agency (JST) , Tempaku , Nagoya , Aichi 468–8502 , Japan

## Abstract

As an alternative to Darwinian evolution relying on catalytic promiscuity, a protein may acquire auxiliary function upon metal binding, thus providing it with a novel catalytic machinery. Here we show that addition of cupric ions to a 6-phosphogluconolactonase **6-PGLac** bearing a putative metal binding site leads to the emergence of peroxidase activity (*k*_cat_ 7.8 × 10^–2^ s^–1^, *K*_M_ 1.1 × 10^–5^ M). Both X-ray crystallographic and EPR data of the copper-loaded enzyme **Cu·6-PGLac** reveal a bis-histidine coordination site, located within a shallow binding pocket capable of accommodating the *o*-dianisidine substrate.

## Abstract 2

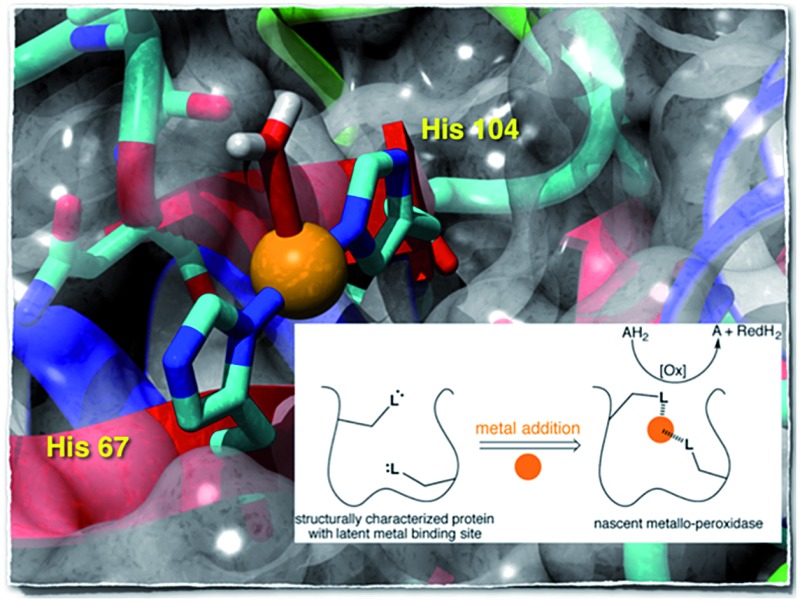
Adding a metal cofactor to a protein bearing a latent metal binding site endows the macromolecule with nascent catalytic activity.

## Introduction

Metal ions are present in nearly half of the characterized proteome,^1^,^2^ and metalloenzymes catalyze some of nature's most challenging reactions.^3^ This versatility has inspired a number of different strategies to create artificial metalloenzymes in the past twenty years.^4^–^6^ Guided either by computation or intuition,^4^–^7^ artificial metalloenzyme design by-and-large relies on the *de novo* introduction of a metal-binding site within a protein scaffold or peptide.^8^–^13^ For this purpose, various strategies have been pursued including covalent-, supramolecular- and dative anchoring.^4^–^6^ The latter approach may rely on incorporating either natural- or non-natural aminoacids as ligands (*i.e.* bipyridinylalanine *etc.*).^14^–^16^ Alternatively, metal-substitution within a metalloenzyme can lead to novel catalytic activity.^17^–^21^ Eventually, the nascent catalytic activity may be further improved by directed evolution strategies.^22^–^25^

**Fig. 1:**
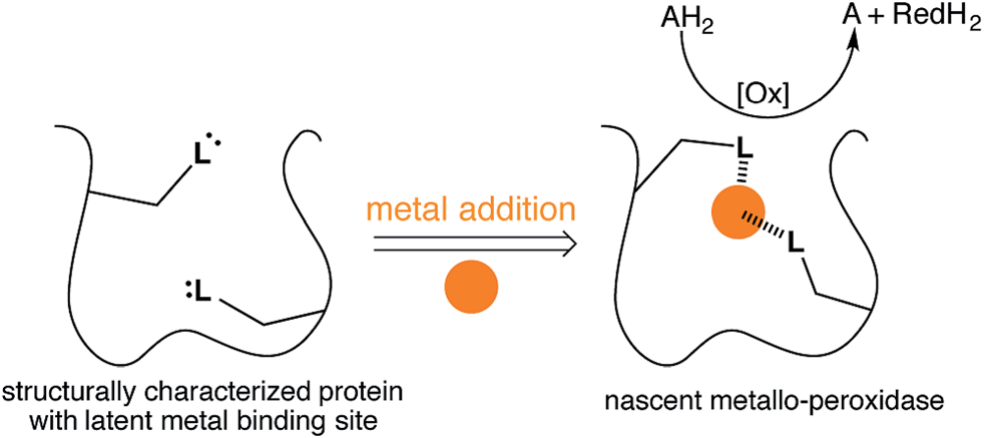
Emergent enzymatic activity resulting from metal acquisition by a protein bearing a latent metal binding motif.

To complement these efforts and in an “enzyme tinkering spirit”,^26^,^27^ we hypothesized that non-metal containing proteins may, through the course of random mutations, evolve a putative metal binding site. Upon acquisition of a metal, nascent catalytic activity may arise, Fig. 1. As with enzyme promiscuity,^28^–^30^ if the newly acquired asset provides a competitive advantage to the cell,^31^,^32^ it may evolve to a highly efficient metalloenzyme.

To test this hypothesis we employed the “Search for Three dimensional Atom Motifs in Protein Structure” (STAMPS) algorithm,^33^ which allows to identify proteins by a systematic search in the protein databank (PDB) for structures possessing a given motif in a topology similar to that adopted by the functional motif in a reference protein. Using this approach, we recently reported on the *in silico* identification of non-metallated two-histidine one-carboxylate metal binding motifs within the structurally characterized proteins of the PDB.^34^ Herein we report our efforts to valorise the *in silico* study by creating an artificial metallo-peroxidase upon addition of a metal cofactor to a protein harboring a latent mononuclear metal binding site, Fig. 1.

## Results and discussion

The proteins bearing a latent non-metallated two-histidine one-carboxylate motif (HHD/E, hereafter) were identified previously using the STAMPS algorithm. For this study, we selected seven scaffolds bearing an HHD/E motif located within a binding pocket predisposed to bind metals.^33^,^34^ The search was expanded to include HHN/Q motifs, revealing six additional potential metal binders following a single point mutation. In total, six of the thirteen cloned proteins could be overexpressed in *E. coli* and purified using a Strep-tag II (Fig. S1–S4†).

**Fig. 2:**
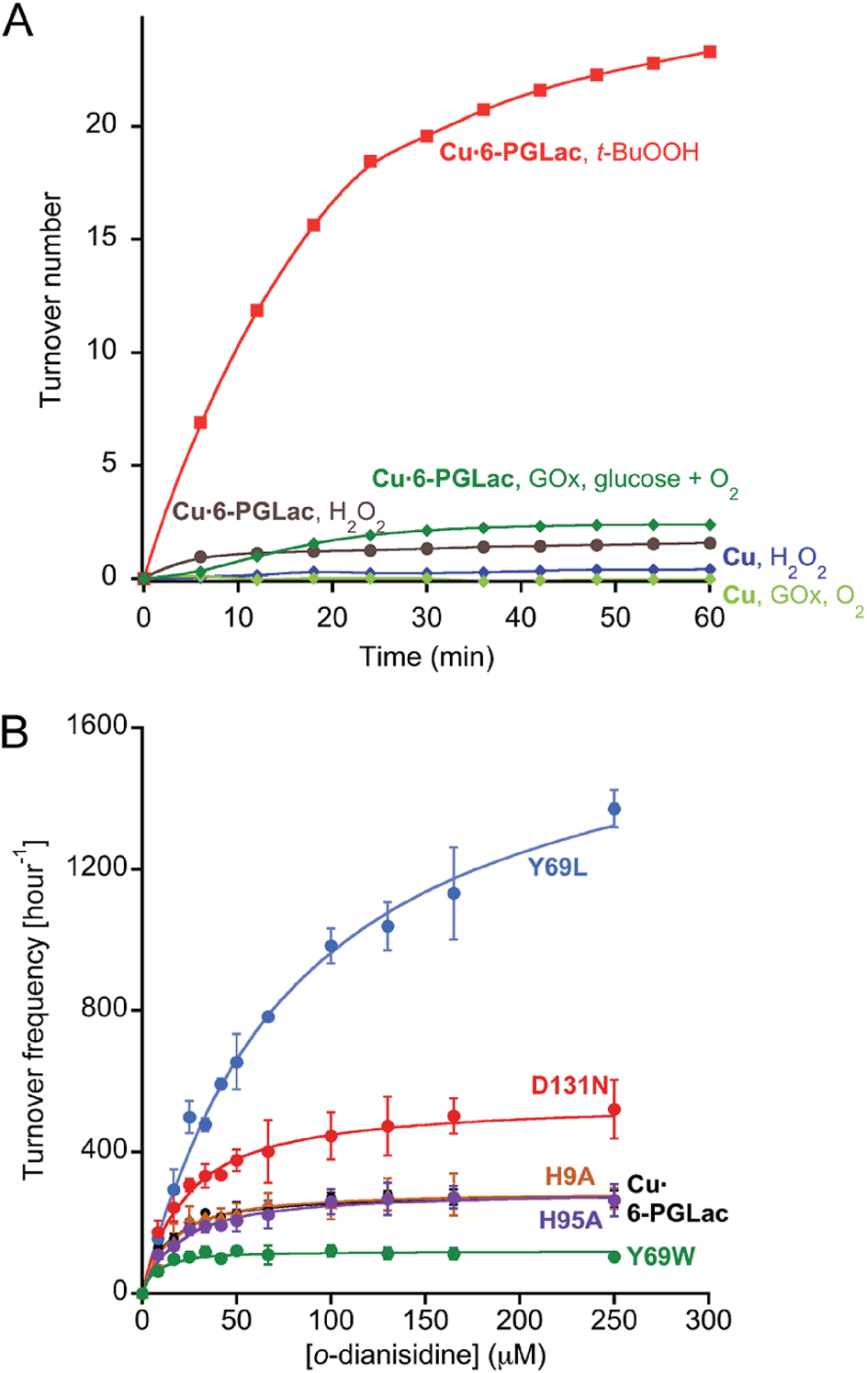
(A) Identification of nascent peroxidase activity resulting from copper addition to a lactonase using H_2_O_2_ (brown) and *t*-BuOOH (red) as oxidizing agent. In the presence of glucose oxidase (GOx) and glucose, **Cu·6-PGLac** displays peroxidase activity (green). None of the other metals tested (including Ni^2+^, Mn^2+^, Fe^2+^, Co^2+^, VO^2+^) displayed significant peroxidase activity in the presence of *t*-BuOOH and **6-PGLac** (TON < 2 after 60 min, Fig. S6A†). (B) Michaelis–Menten saturation kinetics for the oxidation of *o*-dianisidine with *t*-BuOOH (6.6 mM) for selected mutants (1.25 μM) in the presence of copper (4.5 μM), [*o*-dianisidine] = 8–250 μM in MES-buffer (pH 6.5, 50 mM, 150 mM NaCl, 25 °C). Measured data (symbols); fitted data (solid lines). The minimal background reaction caused by free copper was subtracted from the raw data for fitting purposes (Fig. S12B†).

These wild-type proteins or their single mutant isoforms (pdb code: 3D53, ; 2F99, ; 1JSY, ; 1FHI (bearing a Q83E mutation), ; 1MEJ (bearing a N106D mutation), ; 3OC6 (bearing an N131D), see Table S1†) were tested for their peroxidase activity in the presence of various transition metal salts including VOSO_4_, MnCl_2_, FeCl_2_, CoSO_4_, NiCl_2_ and CuSO_4_. For this purpose, *o*-dianisidine (0.75 mM) and hydrogen peroxide (1.5 mM) were added to a buffered solution containing the protein (20 μM) and the metal salt (25 μM). The appearance of the quinone-diimine oxidation product was monitored at 460 nm.^35^ Gratifyingly, this screen revealed one hit: in the presence of CuSO_4_, 6-phosphogluconolactonase bearing an N131D mutation (**Cu·6-PGLac** hereafter) catalyzes the oxidation of *o*-dianisidine (Fig. 2A, S5 and S6†).

**Fig. 3:**
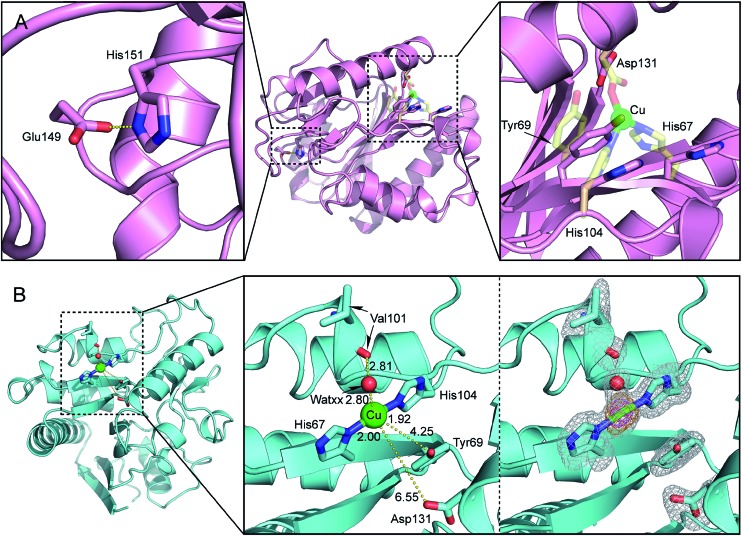
(A) Close-up view of the putative metal binding site of 6-phosphogluconolactonase **6-PGLac** (PDB code ; 4TM8); the native hydrolytic site (left), located 26 Å away from the putative metal binding site (right). The protein main chain is displayed as ribbon and key amino acid residues are highlighted as sticks (the putative facial triad motif was metallated manually *in silico* and is displayed as transparent yellow sticks); (B) close-up view of the Cu1 binding site in **Cu·6-PGLac** (PDB code ; 4TM7, 2Fo – Fc (gray) and anomalous difference Fourier (orange and magenta) maps contoured at 1*σ*, 5*σ*, and 15*σ*, respectively): Cu1–Nδ(His67), 2.00 Å; Cu1–Nε(His104), 1.92 Å; Cu1–WatXX, 2.80 Å; Nδ(His67)–Cu1–WatXX, 94.2°; Nε(His104)–Cu1–WatXX, 95.8°; and (His67)–Cu1–Nε(His104), 170.0°.

Inspection of the X-ray structure of the putative 6-phosphogluconolactonase (**6-PGLac**, apo form) at 1.81 Å reveals a Rossmann fold annotated to the glucosamine-6-phosphate isomerases/6-phosphogluconolactonase family (PF01182) in PFAM database, with the hydrolytic Glu149–His151 dyad and the latent metal binding site 26 Å apart, (Fig. 3A and Table S4†). The alleged 6-phosphogluconolactonase activity^36^ was confirmed by ^31^P NMR, both in the absence and in the presence of cupric ions, emphasizing the moonlighting nature of this emergent peroxidase activity, Fig. S7.† 29 Among the PF01182 family proteins, the triad residues (His67, His104, and Asn(Asp)131) are *not* conserved, thus suggesting that this potential metal binding motif is not functionally relevant (Fig. S8†).

No peroxidase activity was observed upon substitution of hydrogen peroxide by the dioxygen/ascorbate couple. However, the enzyme cascade consisting of glucose oxidase and **Cu·6-PGLac** restores catalytic activity in the presence of glucose, dioxygen and *o*-dianisidine (Fig. 2A and S6B†). Significant rate enhancement was achieved using *t*-butyl hydroperoxide (*t*-BuOOH) instead of H_2_O_2_ as oxidant (Fig. 2A). The peroxidation of catechol and guaiacol by **Cu·6-PGLac** was investigated (Fig. S5†). No peroxidation product was detected for guaiacol with either H_2_O_2_ or *t*-BuOOH as oxidant. **Cu·6-PGLac** catalyzed the peroxidation of catechol. However, the background peroxidation with CuSO_4_ alone was significant, which contrasts to both *o*-dianisidine and guaiacol.

To gain structural insight into the metalloenzyme activity, **6-PGLac** crystals (*vide supra*) were soaked with a mother liquor containing excess CuSO_4_. This procedure however did not yield suitable diffraction data. Thus, **6-PGLac** was co-crystallized with 3 mM CuSO_4_ followed by soaking with 12 mM CuSO_4_ (**Cu·6-PGLac**). The X-ray structure was refined to a resolution of 1.39 Å (Fig. 3B, Table S4 and Fig. S9†). The overall structure is nearly identical to that of **6-PGLac**, apo form (Root Mean Square Deviation (RMSD) = 0.32 over 243 Cα atoms, Fig. 3A and B and S9A†). The structure contains three fully occupied copper ion binding sites with strong anomalous signals (Table S6†): two copper ions are bound to surface histidines and aspartates crosslinking two protein monomers in the crystal (Cu2 is bound to His9 and Asp3′ and Cu3 is bound to His95 and Asp158′ and Asp220′ respectively, see Fig. S9B†). The third copper (Cu1) is located in the putative metal binding site and displays a T-shaped [2 + 1] coordination geometry. The copper is bound to His67 and His104 (at a distance of 2.00 Å and 1.92 Å, respectively) and displays a weak contact with a water molecule (WATxx 2.80 Å), which is held in place *via* a hydrogen bond to the carbonyl oxygen of Val101 (O···O 2.81 Å). This structure also contains further unspecifically bound copper ions in the lactonase active site as well as on the surface (Fig. S9C and Table S6†). However, these binding sites are characterized by low occupancy, as reflected by the height of anomalous difference density peaks, and thus presumably have a far lower binding affinity for copper ions.

The coordination geometry around Cu1 is T-shaped with two *trans*-histidines and a water ligand. Although reminiscent of the Cu-coordination recently reported for the lytic polysaccharide monooxygenase from *Aspergillus oryzae*, it lacks the terminal amine ligand, characteristic of the “histidine brace”.^37^ While two- and three coordinate copper geometries are frequently encountered in metalloenzymes and coordination complexes, earlier transition metals often prefer higher coordination numbers.^3^ We speculate that the lack of peroxidase activity observed with all other metal salts tested in the presence of **6-PGLac** may be caused by the ill-suited low coordination geometry imposed by the putative active site.

The affinity of **6-PGLac** for Cu(ii) was determined using tryptophan-fluorescence quenching. Two dissociation constants could be extracted from the titration profile: *K*_d1_ = 0.83 ± 0.11 μM, *K*_d2_ = 130 ± 3.3 μM; confirming the presence of one tight and weaker copper binding sites (Fig. S13†).

The potentially coordinating oxygen of Asp131 identified by the *in silico* search and of the Tyr69 are located 6.55 Å and 4.25 Å from Cu1 respectively. The low coordination number of Cu1, coupled with the possibility of photoreduction by the X-ray dose, led us to explore whether cupric state may have additional ligands.

In order to investigate the involvement of neighboring amino acids in the coordination of catalytically competent cupric ions, the kinetic saturation profiles of **Cu·6-PGLac** and mutants (Fig. S12†) thereof were determined. The results are summarized in Table 1, Fig. 2B and S12A.† **Cu·6-PGLac** bearing the HHD triad displays Michaelis–Menten behavior with an efficiency of *k*_cat_/*K*_M_ = 6.9 × 10^3^ M^–1^ s^–1^ (Table 1, entry 1). Interestingly, the *K*_M_ value of **Cu·6-PGLac** is comparable to that of the naturally occurring peroxidase (For horseradish peroxidase (HRP), 13 μM).^38^ The *k*_cat_/*K*_M_ however is lower than that of HRP (7.1 × 10^7^ M^–1^ s^–1^),^38^ whereas it's equal to or greater than those of the artificial peroxidases (ferric porphyrin-binding antibody (3.4 × 10^4^ M^–1^ s^–1^)^39^ and ferric porphyrin-binding xylanase (3.7 × 10^2^ M^–1^ s^–1^)^40^). Deletion of the surface histidines confirms that both Cu2 and Cu3 (bound to His9 and His95 respectively) are not catalytically competent. Indeed both **Cu·6-PGLac** H9A and **Cu·6-PGLac** H95A display nearly identical kinetic behavior when compared to **Cu·6-PGLac** (Table 1, entries 2 and 3). Unfortunately, all attempts to mutate two surface histidine residues invariably led to inclusion bodies which could not be renatured. In stark contrast, mutation of either His67 or His104 binding residues into non-coordinating amino acids completely shuts off catalytic activity, (Table 1, entries 4 and 5), confirming that the catalytic metal is indeed located in the cavity flanked with residues His67, His104, Asp131 and Tyr69. Unexpectedly, mutation of Asp131 to either a hydrophobic, a coordinating or a polar amino acid does not affect the catalytic performance. This suggests that this residue does not bind either to cupric- or cuprous ions (Table 1, entries 6–9). Position 69 in contrast has a larger impact on the catalytic performance: **Cu·6-PGLac** Y69L displays a moderately improved *k*_cat_, albeit at the cost of *K*_M_ (Table 1, entries 10–12). Based on this limited mutagenesis study, we are confident that the catalytic performance could be improved significantly with a larger mutagenesis campaign.

**Table 1:** Summary of saturation kinetic data for **Cu·6-PGLac** mutants investigated in this study^*a*^

Entry	Variant	*K*_M_ (μM)	*k*_cat_ × 10^3^ (s^–1^)	*k*_cat_/*K*_M_ (M^–1^ s^–1^)
1	**6-PGLac**	11 ± 3	78 ± 4	(6.9 ± 2) × 10^3^
2	H9A	12 ± 5	80 ± 6	(6.5 ± 3) × 10^3^
3	H95A	17 ± 5	80 ± 6	(4.6 ± 2) × 10^3^
4	H67F	N.D.^*b*^	N.D.^*b*^	N.D.^*b*^
5	H104F	N.D.^*b*^	N.D.^*b*^	N.D.^*b*^
6	D131A	13 ± 5	100 ± 7	(7.7 ± 3) × 10^3^
7	D131E	10 ± 3	78 ± 4	(7.7 ± 2) × 10^3^
8	D131H	32 ± 6	150 ± 9	(4.9 ± 1) × 10^3^
9	D131N	21 ± 6	150 ± 10	(7.2 ± 2) × 10^3^
10	Y69F	36 ± 8	100 ± 7	(2.9 ± 0.7) × 10^3^
11	Y69W	4.8 ± 2	33 ± 2	(6.9 ± 3) × 10^3^
12	Y69L	82 ± 10	490 ± 30	(5.9 ± 0.9) × 10^3^

^*a*^The kinetic measurements were determined in triplicate and fitted using the Michaelis–Menten equation. The minimal background reaction caused by free copper was subtracted from the raw data for fitting purposes.

^*b*^The catalytic activity was too small to be determined (Fig. S12B).

**Fig. 4:**
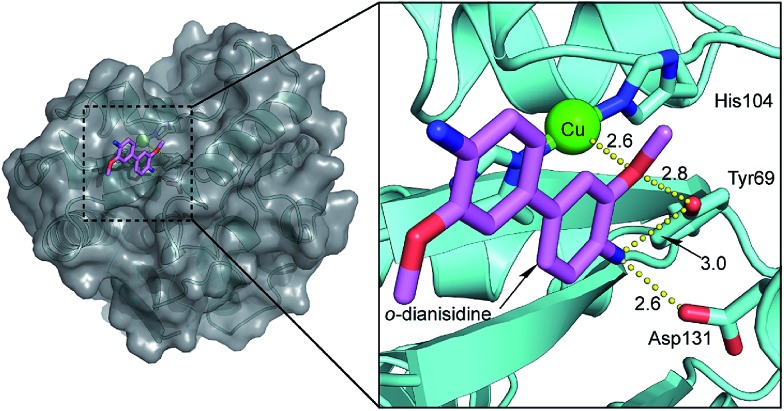
Close-up view of Cu1 site of **Cu·6-PGLac** docked with *o*-dianisidine. Gray, surface representation; the protein main chain is displayed as ribbon and key aminoacid residues as well as *o*-dianisidine are highlighted as sticks.

The involvement of Tyr69 was assessed by performing a docking simulation between **Cu·6-PGLac** and *o*-dianisidine. It revealed one major low-energy conformation with the polar edge of the biaryl-substrate pointing towards the copper active site, Fig. 4. One methoxy oxygen atom of *o*-dianisidine is hydrogen bonded to the oxygen atom of Tyr69 (H_3_CO···O_Y_ 2.8 Å) and interacts with Cu1 (H_3_CO···Cu 2.6 Å). The other hydrogen bonds were observed between nitrogen atom of *o*-dianisidine and the oxygen atom of Tyr69 at a distance of 3.0 Å, and the carboxyl oxygen atom of Asp131 at 2.6 Å.

**Fig. 5:**
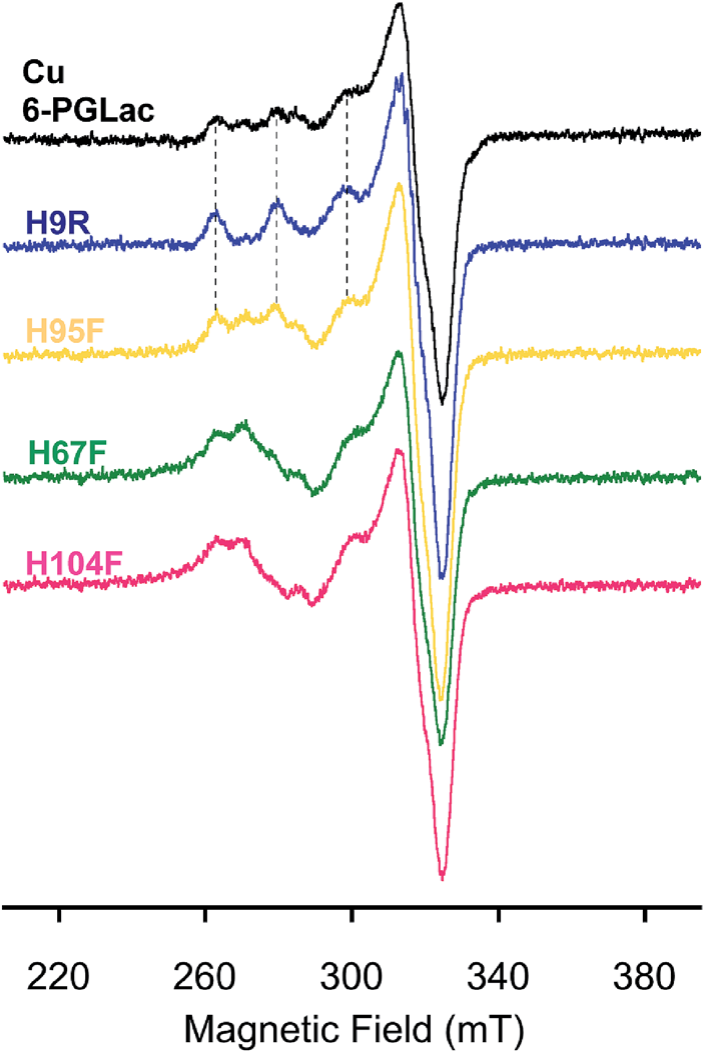
EPR spectra of **Cu·6-PGLac** (black), **Cu·6-PGLac** H9R (blue), **Cu·6-PGLac** H95F (yellow), **Cu·6-PGLac** H67F (green), and **Cu·6-PGLac** H104F (pink) at pH 6.5 (50 mM MES and 150 mM NaCl) at 77 K.

The artificial peroxidase was scrutinized by X-band EPR analysis at 77 K (Fig. 5). The presence of multiple Cu^2+^ species in **Cu·6-PGLac** results in a complicated spectrum in the parallel region (Fig. 5 and S14A†), highlighting the presence of multiple Cu^2+^ binding sites as observed in the crystal structure (Cu1; His67 and His104, Cu2; His9, Cu3; His95). Analysis of histidine-deleted variants revealed that **Cu·6-PGLac** H9R exhibits an axial EPR spectrum, typical of a single Cu^2+^ species (Fig. 5 and S14A†), whereas **Cu·6-PGLac** H95F shows an almost identical spectrum to that of **Cu·6-PGLac**. This suggests that His9 binds to Cu^2+^ more tightly than His95. In contrast, the H67F and H104F single mutants resulted in markedly different spectra (Fig. 5), suggesting that both His67 and His104 are involved in copper coordination in **Cu·6-PGLac**. A reasonable simulation of the spectrum of **Cu·6-PGLac** H9R provided detailed EPR parameters (see Fig. S14C† for further details). Again here, a more detailed analysis was hampered by the formation of inclusion bodies upon mutation of multiple surface histidine residues. Given the parameters of the parallel region (*g*_*z*_ = 2.230 and *A*_*z*_ = 17.7 mT), Peisach–Blumberg analysis^41^ suggests that this Cu^2+^ species is a type 2, reminiscent of the type 2 copper in the multicopper oxidase (*g*_*z*_ ∼ 2.24 and *A*_*z*_ = 13–19 mT).^42^ This copper center bears two nitrogens and one or two water ligand(s) in the equatorial plane in the reported crystal structure.^43^,^44^ Superhyperfine coupling pattern due to the ligating nitrogen atoms is apparent in the perpendicular region (Fig. S14†). Thus, these results suggest that the **6-PGLac** provides the Cu1 ion with a coordination environment suitable for coordination of both the peroxide and the substrate to yield a transient end-on (alkyl-)peroxide copper(ii) species.^45^,^46^ Although, no known natural peroxidase contains a mononuclear copper active site to our knowledge, mononuclear cupric coordination complexes display peroxidase activity and have been shown to operate *via* concerted H atom abstraction with O–O bond scission and subsequent attack at a substrate by a Cu(ii)-oxyl radical (*i.e.* L_*n*_Cu^II^–O˙).^45^–^47^

## Conclusions

The enzyme repurposing strategy contrasts with other artificial metalloenzyme approaches. Indeed, as we demonstrate herein, the latent metal binding site is *present but not metallated* in the wild-type **6-PGLac**. Simple metal salt addition thus suffices to endow a protein with novel catalytic activity, very different from the native activity. Furthermore, this strategy relying upon metal acquisition can be viewed as complementary to enzyme promiscuity, offering a novel means to swiftly acquire enzymatic catalytic activity, vastly different from the native activity.

Although the STAMPS algorithm suggested a possible facial triad coordination, a T-shaped Cu coordination was observed upon Cu(ii) supplementation. We believe that this may be due to the size of the latent metal binding site which allows for significant side chain flexibility: a feature not taken into consideration in the STAMPS algorithm. Gratifyingly, this low-coordination bis-histidine copper geometry combined with the additional surrounding Tyr69 and Asp131 residues provides unique opportunities to fine-tune or alter the emergent peroxidase activity. The compatibility of the artificial metalloenzyme with other enzymes (*e.g.* glucose oxidase) as well as cell lysates suggests that directed evolution strategies may be used to further optimize its performance.

## Supplementary Material

Supplementary information
