# Supplementary material for: Enzyme repurposing of a hydrolase as an emergent peroxidase upon metal binding
Source: Chem Sci. 2015 May 7;6(7):4060–5. doi: 10.1039/c5sc01065a (PMC5707476; doi:10.1039/c5sc01065a)
Supplement: Supplementary file 1 [file SC-006-C5SC01065A-s001.pdf]

Electronic Supplementary Material (ESI) for Chemical Science.
This journal is © The Royal Society of Chemistry 2015

Electronic Supplementary Information (ESI)

for

Enzyme repurposing of a hydrolase as a nascent peroxidase upon metal binding

Nobutaka Fujieda,[a,*] Jonas Schätti,[a] Edward Stuttfeld,[b] Kei Ohkubo,[c] Timm Maier,[b] Shunichi Fukuzumi,[c] and Thomas R. Ward[a,*]

[a]Department of Chemistry, University of Basel, Spitalstrasse 51, CH-4056 Basel, Switerland,
[b]Biozentrum, University of Basel, Klingelbergstr. 50/70, CH-4056 Basel, Switzerland
and
[c]Department of Material and Life Science, Graduate School of Engineering, ALCA, Japan Science and Technology Agency (JST), Osaka University, 2-1 Yamada-oka, Suita, Osaka 565-0871, Japan

Experimental

General. The reagents and the solvents used in this study are commercially available and were used without further purification. UV-visible spectra were recorded on a ND-1000 Nanodrop Spectrophotometer (Nanodrop Technologies Inc.). ESI-MS analysis of the purified proteins was carried out as follows: Reverse phase chromatography was performed on a Dionex Ultimate 3000 System with UV detection at 280 nm. For analytical runs a C4 column (phenomenex Jupiter 5 μm 2 × 50 mm) was used. Proteins were eluted with gradients of solvents A and B (A, acetonitrile containing 0.1% HCOOH; B, H_2O containing 0.1% HCOOH). The HPLC eluents were directed to a maXis 4G mass spectrometer (Bruker Daltonics).

Construction of Plasmids for Selected Protein. The DNA fragments of six proteins used in this study with optimized codons and an N-terminal Strep-tag II for expression in *E. coli* were purchased from Genescript (Table S2). These DNA fragments were cloned into the EcoRV site of pUC57Kan plasmid. The inserted DNA fragments corresponding to each protein genes were amplified with Q5 polymerase (NEB) using a pair of primers (Table S2) from each plasmid. To sub-clone each DNA fragments into the multicloning site of pET26b vector (Novagen) (Fig. S1 and S2), the vector DNA fragment were amplified from pET26b vector using a pair of primers (Table S2), removing *pelB* leader and His-tag regions. Two pieces of fragments were ligated by using directional cloning system, InFusion (Clontech).

Site-directed Mutagenesis. Oligonucleotide-directed mutagenesis experiments were performed on the expression vector containing the DNA encoding **6-PGLac** (Fig. S1). The pairs of about 24-31 base mutagenic oligonucleotides (Table S3) were purchased from Microsynth. Mutagenic inverse PCRs were carried out with these pairs, following DpnI digestion and self-ligation. For the mutants, the absence of undesired mutations in the gene were confirmed by DNA sequence analysis service (Microsynth).

Expression and Purification Methods. The pET26-based expression constructs were transformed into competent *E. coli* BL21(DE3) cells. After incubating for 6 h at 37 °C in LB medium containing 30 μM kanamycin, 2 mL of the pre-culture suspension was added to 1 L of autoinduction medium (ZY5052 medium + 50 μM kanamycin).[1] The culture was incubated in a 5 L baffled Erlenmeyer flask at an appropriate temperature and time (37 °C for protein 1, 4 and 5, 16 h at 37 °C; step-down temperature, 1 h at 36 °C + 3 h at 29 °C + 12 h at 22 °C + 32 h at 15 °C) depending on the proteins (Fig. S3). The cell pellet was washed with 0.9 % aqueous NaCl once and then stored at -20 °C until use. The cell pellet (10 g) was thawed and suspended in 10 fold binding buffer (100 mM Tris-HCl, 150 mM NaCl, pH 8.0), then Lysozyme (0.2 mg/mL), DNase I (0.05 mg/ml) and one protease inhibitor tablet (Complete without EDTA, Roche) were added. Then, the suspension was placed on ice for 45 min, sodium deoxycholate (0.5 mg/mL) was added and the suspension was placed on ice for another 15 min. After centrifugation for 30 min at 15000 × *g*, to remove the cell debris, the supernatant was collected. In order to inhibit biotin to bind to Strep-tactin, streptavidin (1 mg/mL) was added to the supernatant. The Strep-tag II fused protein was purified by affinity chromatography using a 4 × 2 mL Strep-tactin column according to the supplier's manual (Strep-taction superflow high capacity, IBA). An additional wash step was carried out using 10 column volumes of wash buffer (100 mM Tris-HCl, 150 mM NaCl, and 1 mM EDTA, pH 8.0) after allowing the protein to bind to the Strep-tactin. The eluted protein was concentrated to a volume of 1 mL using Vivaspin Turbo 15 (Sartorius) centrifugal concentrators.

Further purification was carried out by size-exclusion chromatography with a Superdex 75 gel permeation column (HiLoad 16/600, GE Healthcare) equilibrated in SEC buffer (20 mM Tris-HCl, 150 mM NaCl, pH 8.0) at a flow rate of 1 ml/min. The purified protein was concentrated and adjusted to 10 mg/ml. The protein samples were kept at -20 °C until use. Protein concentration was determined by measuring the intensity of absorbance at 280 nm.[2]

Screening for Nascent Peroxidase Activity. 5 μL of the metal ion solution (2.5 mM, $FeSO_4$, $NiCl_2$, $MnCl_2$, $CuSO_4$, $ZnCl_2$, $CoSO_4$ or $VOSO_4$) was added to a protein stock solution (0.5 mL, 20 μM) in 20 mM Tris-buffer at pH 8.0 containing 150 mM NaCl. Then, H_2O_2 (7.5 μL, 100 mM) and *o*-dianisidine (5 μL, 25 mM) were added to initiate the oxidation reaction of *o*-dianisidine.

Crystallization of 6-PGLac (Apo-form without $CuSO_4$) and Cu·6-PGLac (6-PGLac with $CuSO_4$). Crystals of both forms were obtained by the sitting drop vapor diffusion method at 20 °C. Crystallization droplets were prepared by mixing the precipitant solution (0.2 μl) and protein solution (0.2 μl). 10 and 25 mg/ml solution were used for without and with $CuSO_4$, respectively. Precipitant contains Mix A (20% polyethylene glycol 1000, 0.2 M Li_2SO_4, and 0.1 M phosphate/citrate (pH 4.2)) and Mix B (22.5% polyethylene glycol 4000, 15% glycerol, 150 mM $(NH_4)_2SO_4$, and 3 mM $CuSO_4$) for without and with $CuSO_4$, respectively. Crystals of **6-PGLac** were grown in 3 days and were cryoprotected in a reservoir solution supplemented with ethylene glycol in a stepwise fashion (ethylene glycol final concentration (v/v) of 10%, 15%, 20% and 25%, 5 min each) and directly transferred into liquid nitrogen. Crystals of **Cu·6-PGLac** were obtained in 4 days and were cryoprotected in a reservoir solution supplemented with 3 mM $CuSO_4$ and 20 mM Tris-HCl (pH 8.0) and 0.15 M NaCl, and ethylene glycol in a stepwise fashion (ethylene glycol final concentration (v/v) of 10%, 15%, 20% and 25%, 5 min each). The crystals were subsequently soaked into the same solutions with 12 mM $CuSO_4$ for 2h and directly transferred into liquid nitrogen to freeze them.

Data Collection of X-Ray Diffraction and Structure Determination. All diffraction data were collected at the PXI-Xo6SA and the PXIII-Xo6DA beamlines at the Swiss light source (SLS) synchrotron facility at the Paul Scherrer Institute (Villigen, Switzerland). X-ray diffraction images were collected on pilatus 2M and 6M detectors equipped with a cryo-system at 100 K using a wavelength of 1.0 Å. The data were processed and scaled with the XDS program package. The data collection and refinement statistics are summarized in Table S4. The two crystal structures of **6-PGLac** were solved by molecular replacement with Phaser using the model of the reported coordinate (PDB code: 3Q6C) as search model. The resulting model covered 243 residues of the one subunit in an asymmetric unit with 243 side chains assigned. The structure model was manually rebuilt with the program COOT[3] followed by refinement calculations with the program Phenix.refine.[4] This procedure was iterated until the model did not further improve. In the structures, Ramachandran analysis with the Molprobity program showed no outliers.[5] The final structure of **6-PGLac** and **Cu·6-PGLac** contains residues Ala0–Leu244 and Ser2–Leu244 respectively. The electron density at the N-terminal Strep-tag II regions was too weak/absent to allow reliable building of residues Trp(-10)–Lys(-3). The missing residues and atoms are summarized in Table S5. The anomalous difference Fourier maps were calculated from the data set of **Cu·6-PGLac** using the Phenix suite. All figures of protein structures were prepared using PyMOL (Version 1.3 Schrödinger, LLC).

Substrate Docking Simulation. The crystal structure coordinations of **Cu·6-PGLac** (PDB code: 4TM7) and *o*-dianisidine (PDB ligand entry: DDJ) were used in the substrate-protein docking

studies. Hydrogens were added to the **Cu·6-PGLac** structures. The *o*-dianisidine was docked into cavity around Cu1 with GOLD Suite. All of the side chains were not allowed to rotate during docking and twenty dockings were visually inspected.

Kinetic Measurements. The kinetic experiments were performed in a 96-well plate reader (Infinite 200, TECAN). All measurements were conducted at room temperature (~ 25 °C). The reaction mixture was prepared by adding $CuSO_4$ (4.9 μL, 500 μM) and each reactant (H_2O_2 (20 μL, 100 mM), *t*-butyl hydroperoxide (20 μL, 100 mM), ascorbate (7.5 μL, 100 mM), and glucose oxidase (6 μL, 1 μM) with glucose (7.5 μL, 2 M)) to protein solution (280 μL, 4 μM) in Tris-buffer (pH 8.0, 20 mM, 150 mM NaCl). For the other metal ions, solution of 500 μM $FeSO_4$, $NiCl_2$, $MnCl_2$, $CuSO_4$, $ZnCl_2$, $CoSO_4$ or $VOSO_4$ were used instead of $CuSO_4$. The reactions were initiated by adding the substrate solution (7.5 μL, 10 mM). The amount of product was calculated using $\varepsilon_{460\,nm} = 11.3\ cm^{-1}\cdot mM^{-1}$, $\varepsilon_{470\,nm} = 26.6\ cm^{-1}\cdot mM^{-1}$ and $\varepsilon_{389\,nm} = 13.7\ cm^{-1}\cdot mM^{-1}$ for *o*-dianisidine,[6, 7] guaiacol[8] and catechol,[9] respectively.

Michaelis-Menten Kinetics. The steady-state kinetic experiments were carried out in 2 mL disposable cuvettes and the absorbance was measured with a Varian Cary 50 Bio UV/Vis Spectrophotometer every 4 sec at room temperature (~ 25 °C). All experiments were performed in triplicates. The reaction mixture was prepared by adding $CuSO_4$ (210 μL, 250 μM) and *t*-butyl hydroperoxide (*t*-BuOOH 800 μL, 100 mM) to the protein solution (12 mL, 1.25 μM) in MES-buffer (pH 6.5, 50 mM, 150 mM NaCl). To trigger the reactions, various amounts of *o*-dianisidine solutions were added (3.3, 6.6, 10, 13, 17, 20, 26 μL of 2.5 mM and 10, 13, 17, 25 μL of 10 mM). The amount of product was determined using the molar extinction coefficient at 460 nm $\varepsilon_{460\,nm} = 11.3\ cm^{-1}\cdot mM^{-1}$.[6, 7] The reaction velocity was calculated from the initial slope in triplicate. Steady-state kinetic parameters (catalytic turnover number (k_{cat}) and Michaelis constant (K_M) were determined by nonlinear fitting of Michaelis-Menten plots. The background reaction caused by free copper was subtracted from the raw data for fitting purposes (Fig. S12B).

Detection of Native Lactonase Activity. The NMR experiments were recorded with a 600 MHz Bruker spectrometer. The detection of the lactonase activity was performed as described previously.[10] 500 μL of a solution containing $NADP^+$ (3 mM), Glucose-6-phosphate (20 mM), $MgCl_2$ (5 mM) and $CuSO_4$ (12 μM) in TEA-buffer (pH 6.7, 1 M) was prepared for the first NMR experiment (t = 0 min). After 10 min a second NMR experiment was done (t = 10 min). Then Glucose-6-phosphate dehydrogenase (final concentration, 2 μM) was added and the NMR experiments were repeated (up to 20 min). Finally, **6-PGLac** (final concentration, 3.5 μM) was added and another NMR experiments were measured (up to 45 min).

Electron Paramagnetic Resonance Spectroscopy. The EPR measurements at 9 GHz were performed with a JEOL JES-FA100 by using highly purified quartz tubes (I.D. 4.0 mm, Radical Research Inc.) with a rubber septum under an N_2 saturated conditions at 77 K. The microwave power and the modulation amplitude were 1 mW and 0.3 mT, respectively. 500 μM $CuSO_4$ was added into 1 mM (final) protein solution. The spectral simulation was carried out using a simulation software, JEOL AniSimu/FA ver 2.0.0. The simulation parameters are given in Fig. S14C.

Fluorescence experiments. Fluorescence quenching measurements were performed at 20 °C with a JASCO FP-6300 according to the ref.[11] The excitation wavelength and spectral bandwidths were 290 nm and 5 nm, respectively. **6-PGLac** (final concentration, 4.0 μM) was

titrated with a concentrated solution of $CuSO_4$ (4 mM) After each addition, the resulting solution was equilibrated for 15 min before performing the fluorescence measurement. The Cu^{2+} concentration was incrementally varied between 0-97 µM. All experiments were performed in triplicate. Using emission intensity at 333 nm, fluorescence change was analyzed by curve fitting according to the two-ligand binding type equation previously reported.[12]

1. F. W. Studier, *Protein Expr. Purif.*, 2005, **41**, 207-234.
2. S. C. Gill and P. H. Von Hippel, *Anal. Biochem.*, 1989, **182**, 319-326.
3. P. Emsley and K. Cowtan, *Acta Crystallogr. D*, 2004, **60**, 2126-2132.
4. P. D. Adams, P. V. Afonine, G. Bunkoczi, V. B. Chen, I. W. Davis, N. Echols, J. J. Headd, L. W. Hung, G. J. Kapral, R. W. Grosse-Kunstleve, A. J. McCoy, N. W. Moriarty, R. Oeffner, R. J. Read, D. C. Richardson, J. S. Richardson, T. C. Terwilliger and P. H. Zwart, *Acta Crystallogr. D*, 2010, **66**, 213-221.
5. V. B. Chen, W. B. Arendall, J. J. Headd, D. A. Keedy, R. M. Immormino, G. J. Kapral, L. W. Murray, J. S. Richardson and D. C. Richardson, *Acta Crystallogr. D*, 2010, **66**, 12-21.
6. K. Okrasa and R. J. Kazlauskas, *Chem. Eur. J.*, 2006, **12**, 1587-1596.
7. K. X. Huang, X. H. Wu and H. B. Xu, *Biol. Trace Ele. Res.*, 2001, **83**, 83-90.
8. D. R. Doerge, R. L. Divi and M. I. Churchwell, *Anal. Biochem.*, 1997, **250**, 10-17.
9. G. Albarran, W. Boggess, V. Rassolov and R. H. Schuler, *J. Phys. Chem. A*, 2010, **114**, 7470-7478.
10. E. Miclet, V. Stoven, P. A. M. Michels, F. R. Opperdoes, J. Y. Lallemand and F. Duffieux, *J. Biol. Chem.*, 2001, **276**, 34840-34846.
11. L. Brisson, N. El Bakkali-Taheri, M. Giorgi, A. Fadel, J. Kaizer, M. Reglier, T. Tron, E. H. Ajandouz and A. J. Simaan, *J. Biol. Inorg. Chemistry*, 2012, **17**, 939-949.
12. A. Elalaoui, G. Divita, G. Maury, J. L. Imbach and R. S. Goody, *Eur. J. Biochem.*, 1994, **221**, 839-846.

Table S1 Summary of protein structures hosting latent metal binding sites used in this study for nascent activity assay.

No	PDB codes	Annotated protein function / Original organisms	Carboxylate / Amide	Histidine 1	Histidine 2
1	3D53[a]	Pyrophosphatase / Rickettsia prowazekii	Asp 114	His 76	His 77
2	2F99[b]	Polyketide cyclase AKNH / Streptomyces galilaeus	Asp 121	His 107	His 119
3	1JSY[c]	Bovine arrestin-2 / Bos taurus	Asp 290	His 210	His 353
4	1FHI[d]	Fragile histidine triad protein / Homo sapiens	Glu83 (Gln 83)[f]	His 98	His 96
5	1MEJ[e]	Glycinamide Ribonucleotide Transformylase / Homo sapiens	Asp106 (Asn 106)[f]	His 108	His 137
6	3OC6[a]	Putative 6-phosphogluconolactonase / Mycobacterium semegmatis	Asp131 (Asn131)[f]	His 104	His 67

[a]not published. [b]Kallio, P. *et al. J. Mol. Biol.* **357**, 210-220 (2006). [c]Milano, S. K. *et al. Biochemistry* **41**, 3321-3328 (2002). [d]Pace, H. C., *et al. Proc. Natl. Acad. Sci. USA* **95**, 5484-5489 (1998). [e]Zhang, Y. *et al. Biochemistry* **41**, 14206-14215 (2002). [f]single point mutants bearing a Glu or Asp instead of Gln or Asn to generate a facial triad motif.

Table S2 Oligonucleotides used for the cloning of expression plasmids.

No.	Sequence (5'-3')	Length	Use
1	TAAGATCCGGCTGCTAACAAAGC	23	Forward primer for vector
2	CATATGTATATCTCCTTCTTAAAGTTAAACAA	32	Reverse primer for vector
3	GGAGATATACATATGGCAAGCTGGAGCCACCCGCA	35	Forward primer for insert
4	AGCAGCCGGATCTTAGTTGCGGTCAATGCCTTCTT	35	Reverse primer for insert of protein 1
5	AGCAGCCGGATCTTACGGGCGACGATGTTCGGTT	34	Reverse primer for insert of protein 2
6	AGCAGCCGGATCTTAGCGGTCATTCAGACGCGGCGA	36	Reverse primer for insert of protein 3
7	AGCAGCCGGATCTTATTGAAAGTAAACACGCAGTG	35	Reverse primer for insert of protein 4
8	AGCAGCCGGATCTTATTCTTCTTTGACCCAGCAG	34	Reverse primer for insert of protein 5
9	AGCAGCCGGATCTTACAGCTTCGCCGCCGCCGCTT	35	Reverse primer for insert of protein 6

Table S3 Oligonucleotides used for site-directed mutagenesis of **6-PGLac**[a]

No.	Sequence (5'-3')	Length	Use
1	GCCGATACCGCCGCTCTGGTTGCCGCCGCAG	31	Forward primer for H9X
2	GGCGCGTTCAATGACGGTATCGGACATG	28	Reverse primer for H9A
3	ACGGCGTTCAATGACGGTATCGGACATG	28	Reverse primer for H9R
4	ATTGGTATCCCGCCGGTTAATGTC	24	Forward primer for H95X
5	GGCATCCAGCAGAGCTTCACGTGCTTGTTTG	31	Reverse primer for H95A
6	AAAATCCAGCAGAGCTTCACGTGCTTGTTTG	31	Reverse primer for H95F
7	CTATTGGGGCGATGAACGTTTTGTTCCGCAG	31	Forward primer for H67X
8	ATAAACACCTTCGACCAGTCAATTTCACCTG	31	Reverse primer for H67F
9	GCGATGGCGGCCAGTGACGGTGAATTC	27	Forward primer for H104X
10	AAAGACATTAACCGGCGGGATACCAAT	28	Reverse primer for H104F
11	TTTGATAGCTCTGTCCCGGGCTTCG	25	Forward primer for D131X
12	GTTTGCTGACAGCAGCTGTGCGTAACCTG	29	Reverse primer for D131N
13	TTCTGCTGACAGCAGCTGTGCGTAACCTG	29	Reverse primer for D131E
14	GGCTGCTGACAGCAGCTGTGCGTAACCTG	29	Reverse primer for D131A
15	GTGTGCTGACAGCAGCTGTGCGTAACCTG	29	Reverse primer for D131H
16	ATATGCACCTTCGACCAGTCAATTTCACCTG	31	Reverse primer for Y69X
17	CCTGTGGGGCGATGAACGTTTTGTTCCGCAG	31	Forward primer for Y69L
18	CTTTTGGGGCGATGAACGTTTTGTTCCGCAG	31	Forward primer for Y69F
19	CTGGTGGGGCGATGAACGTTTTGTTCCGCAG	31	Forward primer for Y69W

[a]The mutagenic nucleotides are underlined.

Table S4 Data collection and refinement statistics[a]

	**6-PGLac** (PDB: 4TM8)	**Cu·6-PGLac** (PDB: 4TM7)
Data collection		
Space group	$I4_122$	$P3_212$
Cell dimensions		
a, *b*, *c*, Å	176.30, 176.30, 38.29	42.51, 42.51, 222.70
α, β, γ, °	90, 90, 90	90, 90,120
Resolution, Å	62.33–1.81 (1.92–1.81) [b]	111.4–1.39 (1.47–1.39)
R_{merge}	4.8 (61.2)	4.9 (53.4)
$I / \sigma I$	26.9 (4.0)	15.3 (2.3)
Completeness, %	99.8 (98.8)	99.7 (98.9)
Multiplicity	11.4 (11.1)	4.3 (4.2)
Refinement		
Resolution, Å	55.75–1.81 (1.87–1.81)	74.23 –1.39 (1.41–1.39)
No. reflections (work/free)	26529/1375	85955/4420
R_{work} / R_{free}	0.175/0.200	0.149/0.178
No. atoms		
Protein	1857	1862
Ligand/ion	5	35
Water	102	158
B-factors		
Protein	47.0	22.4
Ligand/ion	50.7	42.4
Water	52.2	37.0
rmsd		
Bond lengths, Å	0.009	0.011
Bond angles, °	1.186	1.266

[a]A single crystal was used for both structures. [b]Values in parentheses are for highest-resolution shell.

Table S5 Missing residues and atoms

**6-PGLac** (PDB: 4TM8)
**Missing residues**
Ala(-11)–Gly(-2)
**Missing atoms**
Met1 (Cγ, Cε, Sδ), Glu33 (Oε1, Oε2), Val136 (Cβ, Cγ1, Cγ2), Glu203 (Oε1, Oε2), Arg231 (Nη1, Nη2)
**Cu·6-PGLac** (PDB: 4TM7)
**Missing residues**
Ala(-11)–Met1
**Missing atoms**
Glu33 (Oε1, Oε2), Glu118 (Cδ, Oε1, Oε2), Val136 (Cγ1, Cγ2), Glu203 (Cδ, Oε1, Oε2), Asp174 (Cγ, Oδ1, Oδ2)

Table S6 Occupancies and isotropic B-factors of copper ions in **Cu·6-PGLac**

	Atom Numbering in PDB (4TM7)	Occupancies	Isotropic B-factors
**Cu1**[a]	**305**	**0.99**	**22.1**
**Cu2**[a]	**306**	**0.94**	**27.0**
**Cu3**[a]	**307**	**0.98**	**24.3**
Cu4	**308**	0.44	37.9
Cu5	**309**	0.17	23.2
Cu6	**310**	0.44	60.7
Cu7	**311**	0.35	39.8
Cu8	**312**	0.32	49.4
Cu9	**313**	0.18	49.5
Cu10	**314**	0.43	56.8
Cu11	**315**	0.22	35.1
Cu12	**316**	0.26	46.4
Cu13	**317**	0.23	38.3
Cu14	**318**	0.21	29.2
Cu15	**319**	0.41	55.7
Cu16	**320**	0.23	33.4

[a]Fully-occupied coppers

>Protein 1 (PDB entry: 3D53)
CTCACTATAG GGGAATTGTG AGCGGATAAC AATTCCCCTC TAGAAATAAT TTTGTTTAAC TTTAAGAAGG AGATATACAT
ATG*GCAAGC*T GGAGCCACCC GCAGTTCGAA AAG*GGTGCC*A TGTTTATCAA GAAGATCAAA GCAAAAGCCA ACAACAATGA
AATCAACGTT ATTATCGAAA TTCCGATGAA CTCAGGTCCG ATCAAATATG AATTTGATAA AGAAAGCGGT GCACTGTTTG
TGGACCGTTT CATGCAGACC ACGATGAGTT ATCCGTGCAA CTACGGTTTC ATTCCGGATA CCCTGTCCAA TGATGGCGAC
CCGGTTGATG TCCTGGTGGT TGCACATCAC CCGGTCGTGC CGGGCAGCGT CATTAAATGT CGCGCCATCG GTGTGCTGAT
GATGGAAGAT GAAAGCGGCC TGGACGAAAA AATTATCGCG GTTCCGACCT CTAAGCTGGA TATTACGTTT GACCATATCA
AAGAACTGGA TGACCTGTGC GAAATGCTGA AAAAGCGTAT CGTCCATTTC TTTGAACACT ACAAAGATCT GGAAAAGGGC
AAGTGGGTGA AGGTTACGGG CTGGGGCGAC AAGGTGAAAG CAGAAACCCT GATTAAAGAA GGCATTGACC GCAAC**TAA**GA
TCCGGCTGCT AACAAAGCCC GAAAGGAAGC TGAGTTGGCT GCTGCCA*CCG* *CTGAGCAATA* *ACTAGC*ATAA CCCCTTGGGG

>Protein 2 (PDB entry: 2F99)
CTCACTATAG GGGAATTGTG AGCGGATAAC AATTCCCCTC TAGAAATAAT TTTGTTTAAC TTTAAGAAGG AGATATACAT
ATG*GCAAGC*T GGAGCCACCC GCAGTTCGAA AAG*GGTGCC*T CGGAACAAAT CGCCGCTGTC CGCCGCATGG TTGAAGCCTA
CAATACGGGC AAGACGGATG ATGTCGCAGA CTACATTCAC CCGGAATATA TGAACCCGGG TACCCTGGAA TTTACGAGCC
TGCGTGGCCC GGAACTGTTC GCGATTAATG TGGCCTGGGT TAAAAAGACC TTTTCTGAAG AAGCGCGTCT GGAAGAAGTG
GGTATCGAAG AACGCGCAGA TTGGGTTCGT GCTCGCCTGG TCCTGTACGG CCGTCACGTC GGTGAAATGG TGGGCATGGC
ACCGACGGGT CGCCTGTTTA GTGGCGAACA GATTCACCTG CTGCATTTCG TTGACGGCAA AATCCATCAC CATCGCGATT
GGCCGGACTA TCAAGGCACC TATCGTCAAC TGGGTGAACC GTGGCCGGAA ACCGAACATC GTCGCCCG**TA** **A**GATCCGGCT
GCTAACAAAG CCCGAAAGGA AGCTGAGTTG GCTGCTGCCA *CCGCTGAGCA* *ATAACTAGC*A TAACCCCTTG GGGCCTCTAA

>Protein 3 (PDB entry: 1JSY)
CTCACTATAG GGGAATTGTG AGCGGATAAC AATTCCCCTC TAGAAATAAT TTTGTTTAAC TTTAAGAAGG AGATATACAT
ATG*GCAAGC*T GGAGCCACCC GCAGTTCGAA AAG*GGTGCC*A TGGGCGACAA AGGCACCCGT GTGTTCAAGA AAGCATCCCC
GAATGGCAAG CTGACCGTCT ACCTGGGCAA GCGTGACTTC GTGGACCATA TTGACCTGGT TGAACCGGTC GATGGCGTGG
TTCTGGTGGA CCCGGAATAT CTGAAAGAAC GTCGCGTTTA CGTCACCCTG ACGTGCGCAT TTCGTTATGG CCGCGAAGAC
CTGGATGTTC TGGGTCTGAC CTTTCGCAAA GACCTGTTTG TGGCAAACGT TCAGTCATTC CCGCCGGCTC CGGAAGATAA
AAAGCCGCTG ACGCGTCTGC AAGAACGCCT GATTAAAAAG CTGGGCGAAC ACGCGTATCC GTTTACCTTC GAAATCCCGC
CGAATCTGCC GTGTAGCGTG ACGCTGCAGC CGGGTCCGGA AGATACCGGC AAGGCCTGCG GTGTTGACTA TGAAGTCAAA
GCGTTTTGTG CCGAAAACCT GGAAGAAAAG ATTCATAAAC GTAATTCTGT CCGCCTGGTG ATCCGTAAAG TTCAATACGC
ACCGGAACGT CCGGGTCCGC AGCCGACCGC TGAAACCACG CGTCAATTCC TGATGAGCGA TAAGCCGCTG CACCTGGAAG
CATCTCTGGA TAAAGAAATT TATTACCATG GTGAACCGAT CTCAGTGAAC GTTCACGTCA CCAACAACAC GAACAAGACC
GTGAAAAAGA TTAAGATCTC GGTTCGTCAG TATGCGGATA TTTGCCTGTT TAACACCGCC CAATACAAAT GTCCGGTTGC
AATGGAAGAA GCTGATGACA CGGTCGCTCC GAGCTCTACC TTTTGCAAAG TTTATACCCT GACGCCGTTC CTGGCAAACA
ATCGTGAAAA ACGCGGCCTG GCTCTGGATG GTAAACTGAA GCATGAAGAC ACGAACCTGG CAAGTTCCAC CCTGCTGCGT
GAAGGTGCCA ATCGTGAAAT CCTGGGTATT ATCGTGAGTT ACAAAGTGAA GGTTAAACTG GTCGTGTCCC GCGGCGGTCT
GCTGGGTGAC CTGGCGTCAT CGGATGTCGC CGTGGAACTG CCGTTTACGC TGATGCACCC GAAGCCGAAA GAAGAACCGC
CGCATCGTGA AGTCCCGGAA CACGAAACGC CGGTGGATAC CAACCTGATT GAACTGGACA CCAATGATGA CGATATCGTG
TTTGAAGATT TCGCCCGTCA GCGCCTGAAA GGTATGAAGG ATGATAAAGA AGAAGAAGAA GATGGCACGG GTTCGCCGCG
TCTGAATGAC CGC**TAA**GATC CGGCTGCTAA CAAAGCCCGA AAGGAAGCTG AGTTGGCTGC TGCCA*CCGCT* *GAGCAATAAC*

>Protein 4 (PDB entry: 1FHI bearing Q83E mutation)
CTCACTATAG GGGAATTGTG AGCGGATAAC AATTCCCCTC TAGAAATAAT TTTGTTTAAC TTTAAGAAGG AGATATACAT
ATG*GCAAGC*T GGAGCCACCC GCAGTTCGAA AAG*GGTGCC*A TGTCATTCCG TTTTGGTCAA CACCTGATTA AGCCGAGCGT
CGTCTTCCTG AAAACCGAAC TGTCCTTCGC CCTGGTCAAC CGCAAGCCGG TGGTTCCGGG TCACGTTCTG GTCTGCCCGC
TGCGTCCGGT CGAACGTTTT CATGATCTGC GTCCGGACGA AGTGGCAGAC CTGTTTCAGA CCACGCAACG CGTTGGCACC
GTCGTGGAAA AACATTTTCA CGGTACCAGC CTGACGTTCT CTATGGAAGA TGGCCCGGAA GCGGGTCAGA CGGTGAAACA
TGTGCACGTT CATGTCCTGC CGCGTAAGGC CGGTGACTTT CACCGCAACG ATAGTATCTA CGAAGAACTG CAAAAGCATG
ATAAGGAAGA CTTCCCGGCG TCGTGGCGTT CGGAAGAAGA AATGGCTGCG GAAGCGGCGG CACTGCGTGT TTACTTTCAA
TAAGATCCGG CTGCTAACAA AGCCCGAAAG GAAGCTGAGT TGGCTGCTGC CA*CC*GCTGAG CAATAACTAG CATAACCCCT

>Protein 5 (PDB entry: 1MEJ bearing N106D mutation)
CTCACTATAG GGGAATTGTG AGCGGATAAC AATTCCCCTC TAGAAATAAT TTTGTTTAAC TTTAAGAAGG AGATATACAT
ATG*GCAAGC*T GGAGCCACCC GCAGTTCGAA AAG*GGTGCC*G CTCGTGTCGC CGTCCTGATT AGTGGTACGG GCTCCAACCT
GCAAGCTCTG ATTGACTCAA CGCGTGAACC GAACTCGTCC GCACAGATTG ATATCGTGAT TTCGAACAAA GCGGCCGTTG
CAGGCCTGGA CAAGGCAGAA CGTGCAGGTA TCCCGACCCG TGTTATCAAC CATAAGCTGT ACAAGAACCG TGTTGAATTC
GATTCCGCGA TCGACCTGGT CCTGGAAGAA TTCTCAATCG ATATTGTGTG CCTGGCCGGC TTTATGCGCA TCCTGAGCGG
TCCGTTCGTG CAGAAATGGA ACGGCAAGAT GCTGGACATT CATCCGAGCC TGCTGCCGTC TTTTAAAGGT AGTAATGCAC
ACGAACAAGC TCTGGAAACC GGCGTGACCG TTACGGGTTG TACGGTTCAT TTCGTCGCGG AAGATGTTGA CGCCGGCCAG
ATTATCCTGC AAGAAGCCGT CCCGGTGAAA CGTGGTGATA CCGTCGCAAC GCTGAGTGAA CGCGTGAAAC TGGCTGAACA
CAAGATTTTT CCGGCAGCTC TGCAACTGGT CGCATCGGGC ACGGTCCAAC TGGGTGAAAA CGGCAAAATC TGCTGGGTCA
AAGAAGAA**TA** **A**GATCCGGCT GCTAACAAAG CCCGAAAGGA AGCTGAGTTG GCTGCTGCCA *CCGCTGAGCA* *ATAACTAGC*A

>Protein 6 (PDB entry: 3OC6 bearing N131D mutation)
CTCACTATAG GGGAATTGTG AGCGGATAAC AATTCCCCTC TAGAAATAAT TTTGTTTAAC TTTAAGAAGG AGATATACAT
ATG*GCAAGC*T GGAGCCACCC GCAGTTCGAA AAG*GGTGCC*A TGTCCGATAC CGTCATTGAA CGCCACGCCG ATACCGCCGC
TCTGGTTGCC GCCGCAGGTG ACCGTCTGGT TGATGCTATC TCGAGCGCAA TTGGTGAACG CGGCCAGGCT ACCATCGTTC

TGACGGGCGG TGGCACCGGT ATTGGCCTGC TGAAACGTGT GCGCGAACGT TCAGGTGAAA TTGACTGGTC GAAGGTGCAT
ATCTATTGGG GCGATGAACG TTTTGTTCCG CAGGATGACG ATGAACGCAA CGACAAACAA GCACGTGAAG CTCTGCTGGA
TCATATTGGT ATCCCGCCGG TTAATGTCCA CGCGATGGCG GCCAGTGACG GTGAATTCGG TGACGATCTG GAAGCAGCTG
CAGCAGGTTA CGCACAGCTG CTGTCAGCAG ACTTTGATAG CTCTGTCCCG GGCTTCGATG TGCATCTGCT GGGTATGGGT
GGCGAAGGCC ACGTCAACTC CCTGTTTCCG GACACGGATG CCGTTCGCGA AACCGAACGT CTGGTGGTTG GTGTGAGTGA
TTCCCCGAAA CCGCCGCCGC GTCGCATTAC CCTGACGCTG CCGGCAGTTC AAAATAGCCG CGAAGTCTGG CTGGTCGTGT
CTGGCGAAGC TAAGGCGGAT GCAGTGGCAG CTGCAGTTGG TGGCGCAGAC CCGGTGGATA TCCCGGCCGC AGGCGCAGTT
GGTCGTGAAC GCACGGTTTG GCTGGTTGAT GAAGCGGCGG CGGCGAAGCT G**TAA**GATCCG GCTGCTAACA AAGCCCGAAA
GGAAGCTGAG TTGGCTGCTG CCA*CCGCTGA GCAATAACTA GC*ATAACCCC TTGGGGCCTC TAAACGGGTC TTGAGGGGTT

Fig. S1 The DNA sequence of multi-cloning site in expression plasmid for the production of Strep-tag II fused proteins 1-6. The codon-optimized DNA sequences of the encoding proteins 1-6 are highlighted in pink, Strep-tag II gene is in blue, and linker regions are in gray and italic. Initiation and termination codons are in bold.

>Protein 1 (PDB entry: 3D53)
MASWSHPQFEKGAMFIKKIKAKANNNEINVIIEIPMNSGPIKYEFDKESGALFVDRFMQTTMSYPCNYGFIPDTLSND
GDPVDVLVVA**HH**PVVPGSVIKCRAIGVLMMEDESGLDEKIIAVPTSKL**D**ITFDHIKELDDLCEMLKKRIVHFFEHYKD
LEKGKWVKVTGWGDKVKAETLIKEGIDRN

>Protein 2 (PDB entry: 2F99)
MASWSHPQFEKGASEQIAAVRRMVEAYNTGKTDDVADYIHPEYMNPGTLEFTSLRGPELFAINVAWVKKTFSEEARLE
EVGIEERADWVRARLVLYGRHVGEMVGMAPTGRLFSGEQI**H**LLHFVDGKIHH**H**R**D**WPDYQGTYRQLGEPWPETEHRRP

>Protein 3 (PDB entry: 1JSY)
MASWSHPQFEKGAMGDKGTRVFKKASPNGKLTVYLGKRDFVDHIDLVEPVDGVVLVDPEYLKERRVYVTLTCAFRYGR
EDLDVLGLTFRKDLFVANVQSFPPAPEDKKPLTRLQERLIKKLGEHAYPFTFEIPPNLPCSVTLQPGPEDTGKACGVD
YEVKAFCAENLEEKIHKRNSVRLVIRKVQYAPERPGPQPTAETTRQFLMSDKPLHLEASLDKEIYY**H**GEPISVNVHVT
NNTNKTVKKIKISVRQYADICLFNTAQYKCPVAMEEADDTVAPSSTFCKVYTLTPFLANNREKRGLAL**D**GKLKHEDTN
LASSTLLREGANREILGIIVSYKVKVKLVVSRGGLLGDLASSDVAVELPFTLM**H**PKPKEEPPHREVPEHETPVDTNLI
ELDTNDDDIVFEDFARQRLKGMKDDKEEEEDGTGSPRLNDR

>Protein 4 (PDB entry: 1FHI bearing Q83E mutation)
MASWSHPQFEKGAMSFRFGQHLIKPSVVFLKTELSFALVNRKPVVPGHVLVCPLRPVERFHDLRPDEVADLFQTTQRV
GTVVEKHFHGTSLTFSM**E**DGPEAGQTVKHV**H**V**H**VLPRKAGDFHRNDSIYEELQKHDKEDFPASWRSEEEMAAEAAALR
VYFQ

>Protein 5 (PDB entry: 1MEJ bearing N106D mutation)
MASWSHPQFEKGAARVAVLISGTGSNLQALIDSTREPNSSAQIDIVISNKAAVAGLDKAERAGIPTRVINHKLYKNRV
EFDSAIDLVLEEFSIDIVCLAGFMRILSGPFVQKWNGKML**D**I**H**PSLLPSFKGSNAHEQALETGVTVTGCTV**H**FVAEDV
DAGQIILQEAVPVKRGDTVATLSERVKLAEHKIFPAALQLVASGTVQLGENGKICWVKEE

>Protein 6 (PDB entry: 3OC6 bearing N131D mutation, **6-PGLac**)
MASWSHPQFEKGAMSDTVIERHADTAALVAAAGDRLVDAISSAIGERGQATIVLTGGGTGIGLLKRVRERSGEIDWSK
V**H**IYWGDERFVPQDDDERNDKQAREALLDHIGIPPVNV**H**AMAASDGEFGDDLEAAAAGYAQLLSA**D**FDSSVPGFDVHL
LGMGGEGHVNSLFPDTDAVRETERLVVGVSDSPKPPPRRITLTLPAVQNSREVWLVVSGEAKADAVAAAVGGADPVDI
PAAGAVGRERTVWLVDEAAAAKL

Fig. S2 Amino acid sequence of Strep-tag II fused proteins used for the screening of peroxidase activity in this study. The amino acid sequences from candidate proteins are in black, Strep-tag II are in red, linker regions are in green and putative metal binding amino acids are highlighted in blue. Thus, the purified Strep-tag II fused proteins were attached to non-original peptide indicated by italic. For clarity, the *C*-terminal alanine of MASWSHPQFEKGA peptide is assigned as residue 0 (residue 1 for protein 2) unless stated otherwise.

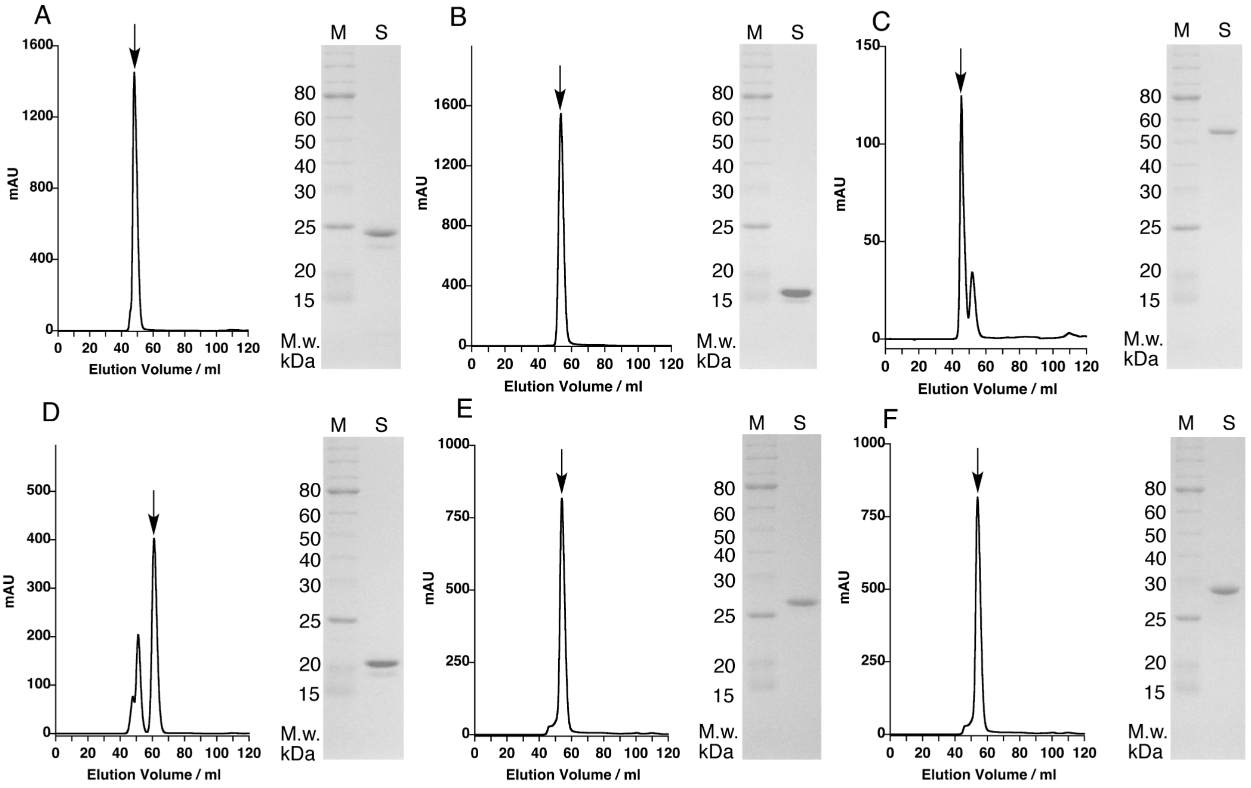

Fig. S3 Size-exclusion chromatograms and SDS-PAGE (14 % acrylamide) analysis of the resulting protein 1 (A), 2(B), 3(C), 4(D), 5(E), and 6(F). M, Marker; S, protein sample. To study the nascent catalytic activity arising from the metallation of the selected proteins, the codon-optimized expression plasmids were created from pET26b vector (See Fig. S1 and S2). After optimization of the culture conditions, protein 1, 4, and 5 were expressed at 37 °C, whereas protein 2, 3, and 6 were expressed under step-down temperature program (36 °C, 29°C, 22°C, and 15°C, see Experimental Section). From *E. coli* cell extracts, the Strep-tag II fused proteins were purified by affinity chromatography with Strep-tactin resin. During this step, the proteins were thoroughly washed with 1 mM EDTA in the wash buffer to remove any metal ions present. The yields of proteins 1, 2, 3, 4, 5, and 6 were about 42, 26, 11, 34, 16, 15 mg per L of medium, respectively. Subsequent purification of the proteins was performed by size-exclusion chromatography (SEC). During this purification step, protein 3 and 4 eluted as two peaks suggesting the possible existence of the two different tertiary structures. The arrows indicate the collected peaks. The homogeneity of isolated proteins was confirmed by SDS-PAGE.

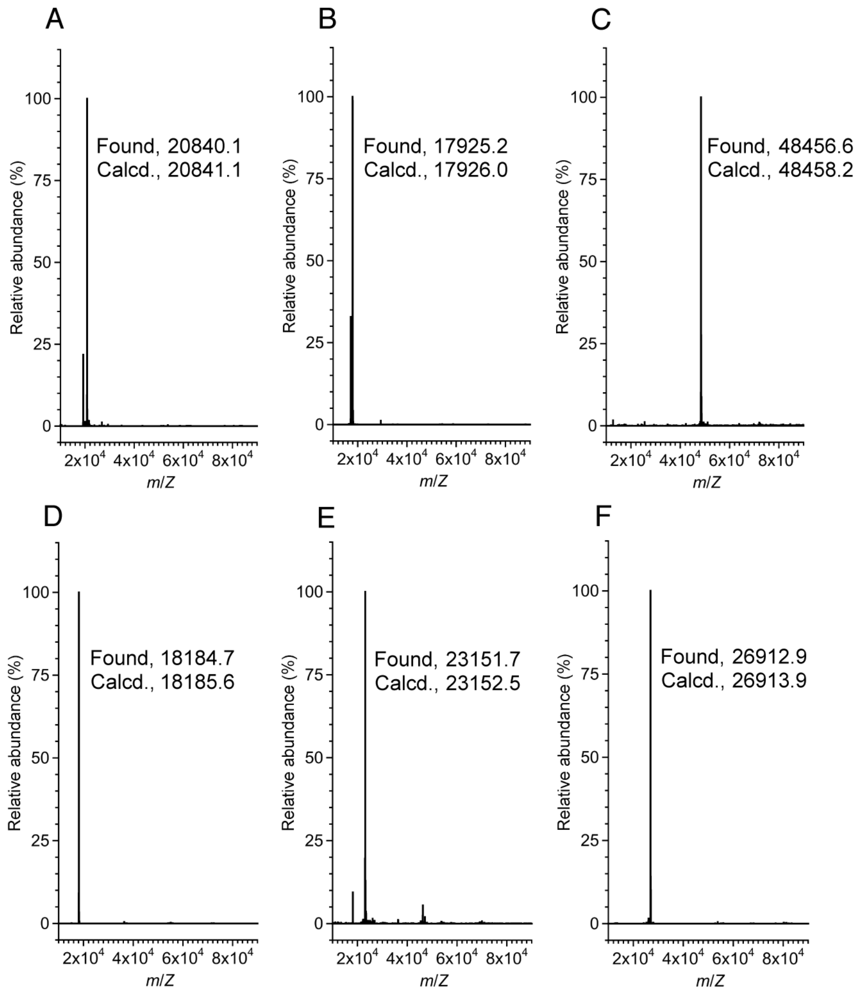

Fig. S4 Deconvoluted ESI-MS spectra of proteins 1 (A), 2 (B), 3 (C), 4 (D), 5 (E), and 6 (F) proteins used in this study. The determined masses are almost identical to calculated mass from the amino acid sequence without initial Met (Fig. S2).

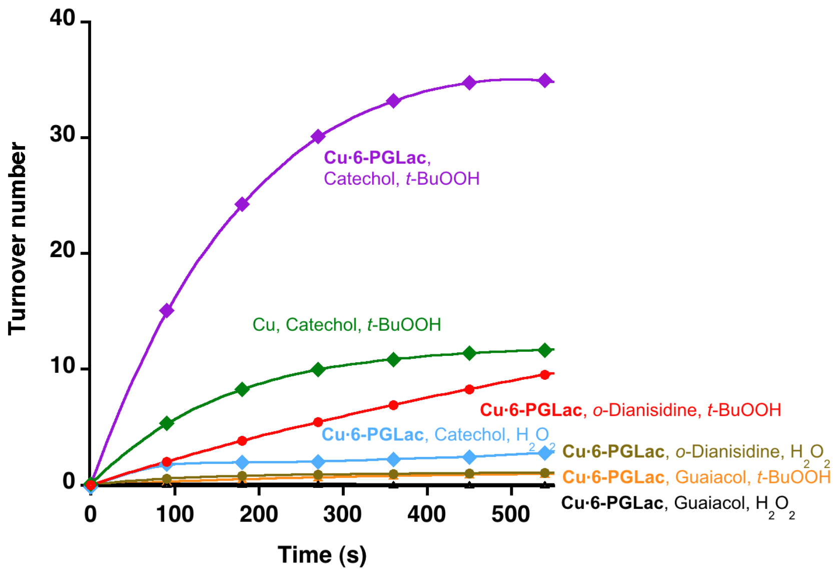

Fig. S5 Time course of the peroxidation of catechol and guaiacol using **Cu·6-PGLac** (4 µM **6-PGLac**, 4 µM $CuSO_4$, 250 µM substrate (catechol: diamonds; guaiacol: triangles; *o*-dianisidine: circles)). The conversions were determined by monitoring the appearance of diphenoquinone and *o*-quinone at 470 nm and 398 nm, respectively. **Cu·6-PGLac** does not catalyze the oxidation of guaiacol with either H_2O_2 (6.6 mM, black) or *t*-BuOOH (6.6 mM, orange). In contrast, **Cu·6-PGLac** catalyses the peroxidation of catechol with H_2O_2 (6.6 mM, blue) or *t*-BuOOH (6.6 mM, purple). However, the background peroxidation reaction of catechol with *t*-BuOOH is significant (compare green- and purple trace).

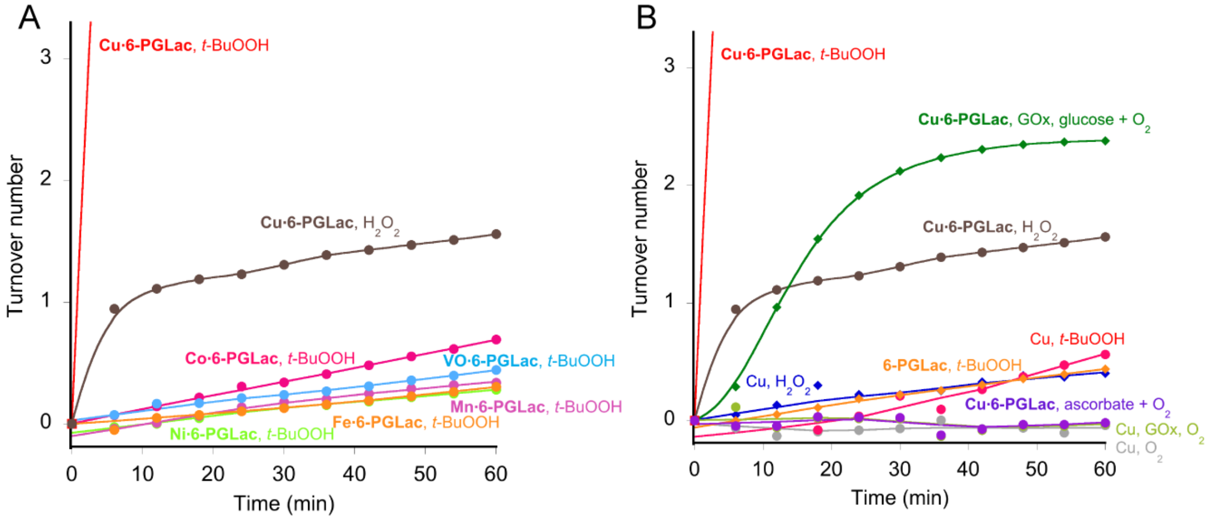

Fig. S6 Time course of *o*-dianisidine oxidation in the presence of various metal ions (A) and oxidizing agents using of **6-PGLac** (B). For comparison, both the **Cu·6-PGLac** catalyzed oxidation of *o*-dianisidine 250 μM with H_2O_2 (6.6 mM, gray trace) and the enzyme cascade consisting of glucose oxidase and **Cu·6-PGLac** (20 nM GOx, 4 μM **6-PGLac**, 4 μM $CuSO_4$, 50 mM glucose, 250 μM *o*-dianisidine, green trace) are displayed. None of the reaction without **6-PGLac** or Cu^{2+} ion proceed (TON < 2 after 60 min).

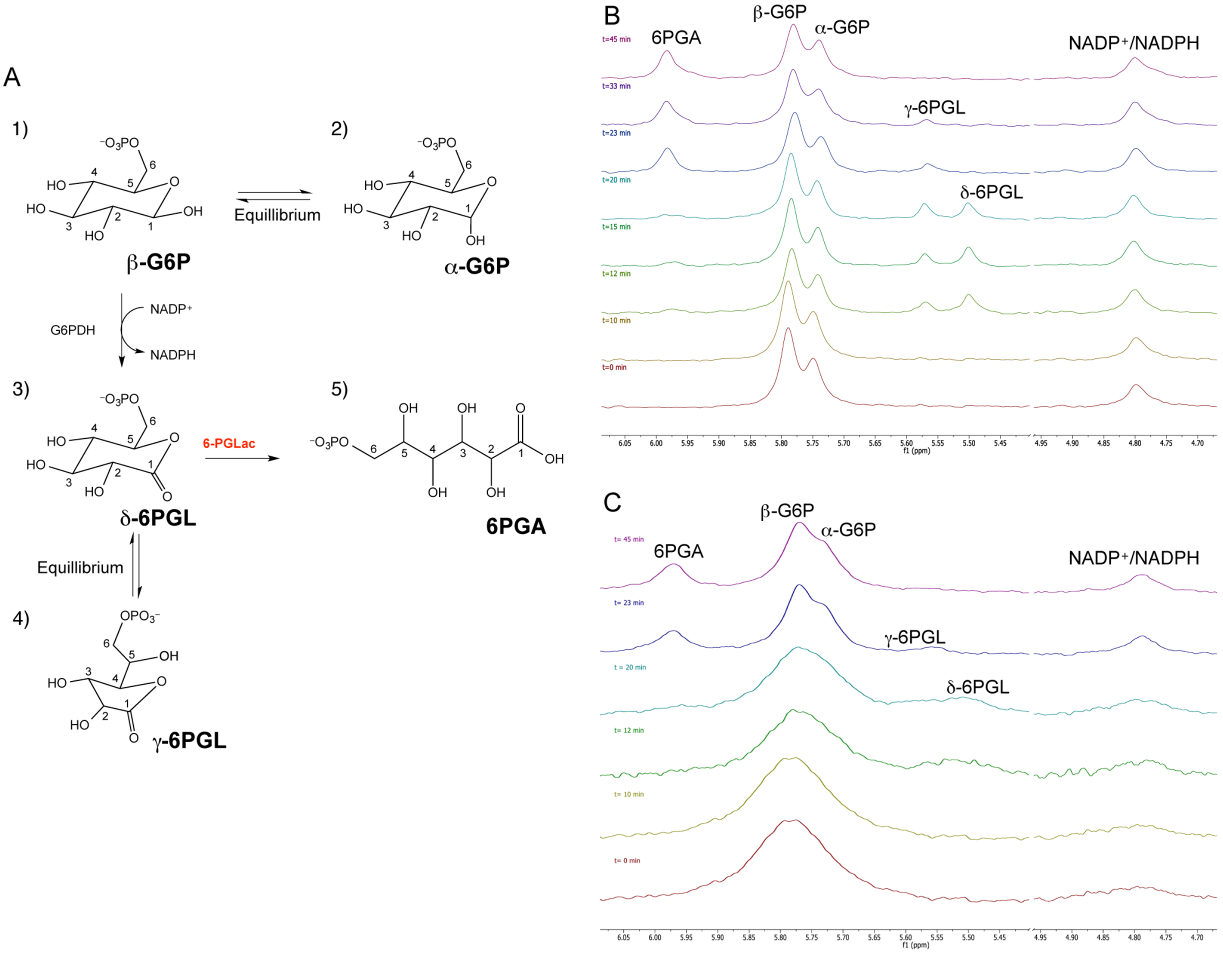

Fig. S7 Schematic representation of the dehydrogenation-coupled lactonase reaction (A) and ^{31}P NMR spectra highlighting the lactonase activity of **6-PGLac** in the absence (B) and in the presence of Cu^{2+} ion (C). During the measurement, a peak shift for the terminal phosphorus of $NADP^+$/NADPH was observed at 4.8 ppm. At the onset, the peaks due to the α-glucose-6-phosphate and β-glucose-6-phosphate (**α-G6P** and **β-G6P**, 5.75 and 5.8 ppm, respectively) as well as the $NADP^+$ signal are visible. During 10 min, no change was observed. Upon addition of Glucose-6-phosphate dehydrogenase (G6PDH, 12 min), new peaks appeared: i) an intense signal at 5.5 ppm and ii) a week signal at 5.55 ppm. These signals are assigned to *δ*-6-phosphogluconolactone and the *γ*-6-phosphogluconolactone, respectively (***δ*-6PGL** and ***γ*-6PGL**). A small signal is also apparent at 6.0 ppm. After 8 min, **6-PGLac** was added. In a following spectrum, the peak of 6-phosphogluconate (**6PGA**, 6.0 ppm) increases rapidly. Accordingly the peaks of the ***δ*-6PGL** and ***γ*-6PGL** gradually decrease. These results demonstrate that **6-PGLac** indeed converts 6-phosphogluconolactone into 6-phosphogluconate.

To investigate the effect of Cu^{2+} ion its native activity, the experiment was repeated in the presence of 12 μM $CuSO_4$ in an NMR-tube. Under these conditions, the conversion from the 6-phosphogluconolactone to 6-phosphogluconate upon addition of **6-PGLac** was still apparent.

Due to peak broadening caused by the presence of paramagnetic Cu^{2+}, the diastereoselectivity (**α-G6P** vs. **β-G6P** and ***δ*-6PGL** vs. ***γ*-6PGL**) could not be determined however. This result clearly demonstrates that the Cu^{2+} binding to **6-PGLac** does not inhibit its native lactonase activity.

```
PDB code                            His67                                Aminoacid No.
3OC6   GGTGIGLLKRVRERSGEIDWSK---VHIYWGDERFVPQDDDERNDKQAREALLDHIGIP-   99
3TX2   GGTGIALLKHLRDVASGLDWTN---VHVFWGDDRYVPKTDPERNAWQAWEALLEHVNFP-   99
3ICO   GGNGIALLRYLSAQAQQIEWSK---VHLFWGDERYVPEDDDERNLKQARRALLNHVDIP-  109
3NWP   GSTPLKLFQLLSMKS--IDWSD---VYITLADERWVEADADASNERLVREHLLQNRASN-   98
3LWD   GSTPKPFFTSLAAKA--LPWAR---VDVTLADERWVTADDADSNARLVRETLLVGPAAE-   94
3LHI   GRSPIAFFNALSQKD--LDWKN---VGITLADERIVPTNHADSNTGLVREYLLKNKAAA-   95
3CSS   GSTPKRLYEELHEKDLALLQQH--AVQFILGDERLLSEDDEQSNFSMATKALLRDVPSSD  100
2J0E   GSTPKMTYARLHDEHLNLLREKR-ALRFFMGDERMVPADSTDSNYNMAREVLLHDIPDDL  100
2RI0   GSTPLELYKEIR----ESHLDFSDMVSINLDEYVGLSADDKQSYAYFMKQNLFAAKPF--   89
2BKV   GGTPEGTYRQLIRLHQTENLSFQNITTVNLDEYAGLSSDDPNSYHFYMNDRFFQHIDS--   94
3HN6   GSSPIGMYKNLIELNKNKKISFQNVITFNMDEYIGIEENHPESYHSFMWNNFFSHIDI--  108
2WU1   GGTPMTTYKALVEMHKAGQVSFKHVVTFNMDEYVGLPKEHPESYYSFMHRNFFDHVDI--   98
1NE7   GSTPLGCYKKLIEYYKNGDLSFKYVKTFNMDEYVGLPRDHPESYHSFMWNNFFKHIDI--   98
1Y89   GSTPKMLFKLLASQPYANDIQWKN-LHFWWGDERCVAPDDAESNYGEANALLFSKINMP-   95
1PBT   GRTPLPVYEKLAEQK--FPWNR---IHFFLSDERYVPLDSDQSNFRNINEVLFSRAKIP-   95
3E15   GKTPIDVYKNIALVKDIKIDTS--KLIFFIIDERYKRDDHKFSNYN-NIKFLFESLKIN-  119
       * .       :                .   :                   ::

                 His104                    Asn(Asp)131
3OC6   ------PVNVHAMAASDGEFGDDLEAAAAGYAQLLSANFDS-------SVPGFDVHLLGM  150
3TX2   ------LRNMHAMPNSESEYGTDLDAAALAYEQLLAANAEPGQ-----DCPAFDVHLLGM  150
3ICO   ------SNQVHPMAASDGDFGGDLDAAALAYEQVLAASAAPGD-----PAPNFDVHLLGM  160
3NWP   ------AKFRGLKNMFSTA-EAGADMAAESLSNFPR---------------PFDVVVLGM  140
3LWD   ------ACFHPLTTDDDTP-EAGVETVAERLESLPW---------------PASAVILGM  130
3LHI   ------AVWIPMVEDGKTETELHPDAVVDYALKHYK---------------QPDVLILGM  130
3CSS   VISIDRRAALATSKDEKGGLDGAWAVAQDYEVKLLNCLPCKQINGTAKSVPVVDIVLLGF  160
2J0E   VFPFDTSAVTPSAEATS---ADAMRVAEAYGKQLASLLPLKSVGEAGPKVPVFDVVLLGL  160
2RI0   ---------KKS--YLPNGLAADLAKETEYYDQILAQY-------------PIDLQILGI  130
2BKV   ---------KPSRHFIPNGNADDLEAECRRYEQLVDSLG------------DTDIQLLGI  130
3HN6   ---------KKENINILNGNASNLKKECEEYEKKIKSFG------------GIMLFVGGI  150
2WU1   ---------PAENINLLNGNAPDIDAECRQYEEKIRSYG------------KIHLFMGGV  140
1NE7   ---------HPENTHILDGNAVDLQAECDAFEEKIKAAG------------GIELFVGGI  140
1Y89   ------AQ-NIHRILGENEPQAEAERFAQAMAHVIPTENG---------TPVFDWILLGV  140
1PBT   ---------SGN--VHYVDTSLPIEKACEKYEREIRSATD-----------QFDLAILGM  130
3E15   ------EKEQLYRPDTSKNIVECVRDYNEKIKNMVKKYT------------KVDIAILGM  160
                                                            : *.
```

Fig. S8 Multiple sequence alignment of aminoacid sequences among PF01182 family protein (glucosamine-6-phosphate isomerases/6-phosphogluconolactonase family) from PDB generated by ClustalW2. The identical and similar residues are marked with * and : (.), respectively. The putative triad residues are shown in red and corresponding residues of other proteins are highlighted with yellow rectangles.

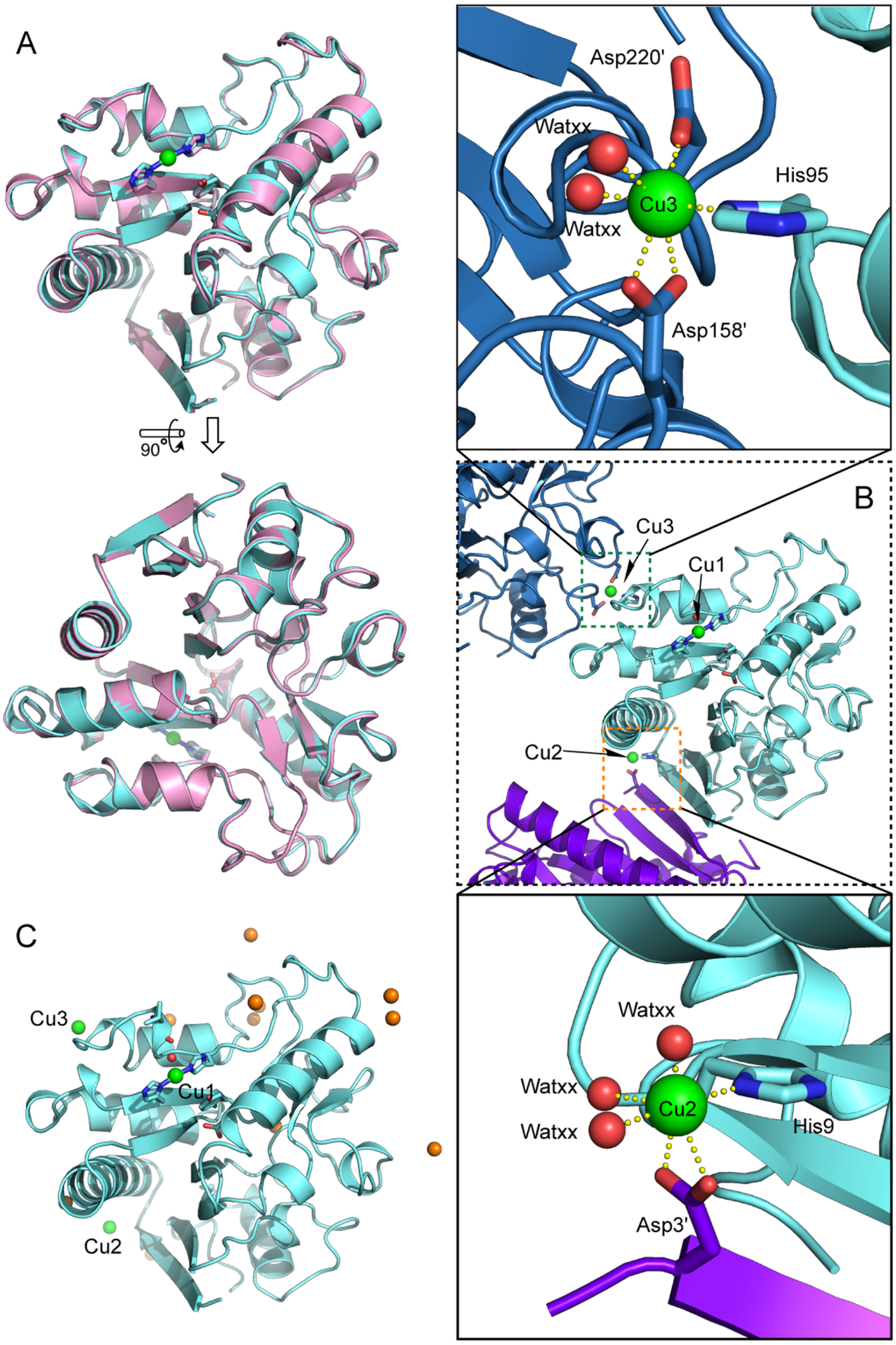

Fig. S9 (A) Superimposed crystal structures of **6-PGLac** (pink) and **Cu·6-PGLac** (Aqua). (B) Close-up views of two fully occupied copper binding sites (green spheres). (C) Other unspecific bound copper binding sites (orange spheres). The crystal structure of **6-PGLac** was determined by molecular replacement using the structure of the reported 6-phosphogluconolactonase (PDB code: 3OC6) as a search model. **6-PGLac** (apo form) was crystallized with one monomer in the crystallographic asymmetric unit (space group $I4_122$) and the final structure was refined to a resolution of 1.81 Å with excellent statistics (Table S4). The protomer consists of 256 amino acid residues deduced from DNA sequence and MS analysis (Fig. S4F) and most of the residues were

well defined in the final electron density, except for some disordered loops in the N-terminal flexible regions (mainly Strep-tag II, Table S5). The overall structure is nearly identical to the reported structure (PDB code: 3OC6, RMSD = 0.57 (243 Cα atoms)).

In the crystal co-crystallized with 3 mM $CuSO_4$ and then soaked with 12 mM $CuSO_4$ (**Cu·6-PGLac**), there is one monomer in the crystallographic asymmetric unit (space group $P3_212$). The final structure was refined to a resolution of 1.39 Å with excellent statistics. In this crystal structure, most of the residues were also well defined in the final electron density, except for some disordered loops in the N-terminal flexible regions (mainly Strep-tag II, Table S5) as it is the case with **6-PGLac**. Three copper ions were identified by strong peaks in anomalous difference Fourier map contoured at 15 σ as threshold value including in the putative metal binding site. The alternative positions for copper binding (Cu2 and Cu3, 22 Å and 32 Å apart from Cu1) were observed in the crystal lattice contacts (Fig. S9B). On the edge of the pseudo β-sheet structure, Cu2 was supported by carboxyl His9 and Asp3' (prime refers to residues from the adjacent protomer). Cu3 was also located on the interface between protomers in the different crystal lattice and supported by H95, Asp158' and Asp220'. Around Cu2, a tight inter-protomer interaction was observed. These two protomers are likely to be held together through 4-hydrogen bonds between Asp3-Arg8 forming an anti-parallel pseudo-β sheet structure on each other. Under physiological conditions, **6-PGLac** was found to exist mainly as a monomer.

Detailed scrutiny of the anomalous difference Fourier map contoured at 3 σ as threshold value, 13 other copper binding sites were found on the protein surface including the lactonase active site (Cu7, Table S6 and Fig S9C). All of these copper ions were > 12 Å far from Cu1 and have much lower occupancies and higher isotropic B-factors (0.299 and 42.7 (average), respectively) as compared to those of the three copper binding sites described above (0.970 and 24.5 (average), respectively). The detailed B-factors of each copper are summarized in Table S6.

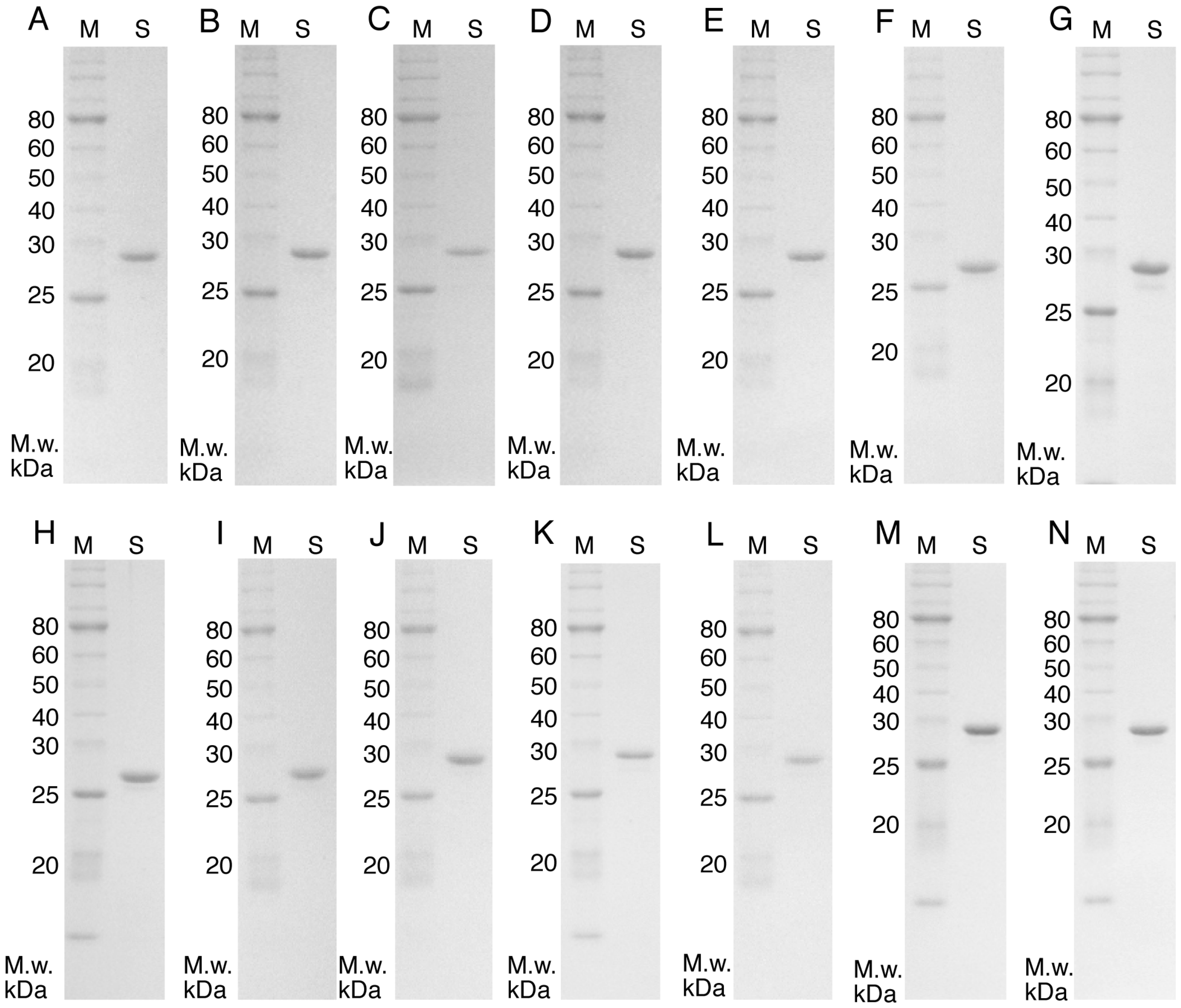

Fig. S10 SDS-PAGE (14 % acrylamide) analysis of the 6-phosphogluconolactonase (**6-PGLac**) isoforms used in this study. A) **6-PGLac** (same data to Fig. S3F); B) H9A; C) H95A; D) H67F; E) H104F; F) D131A; G) D131E; H) D131H; I) D131N; J) Y69F; K) Y69W; L) Y69L; M) H9R; N) H95F.

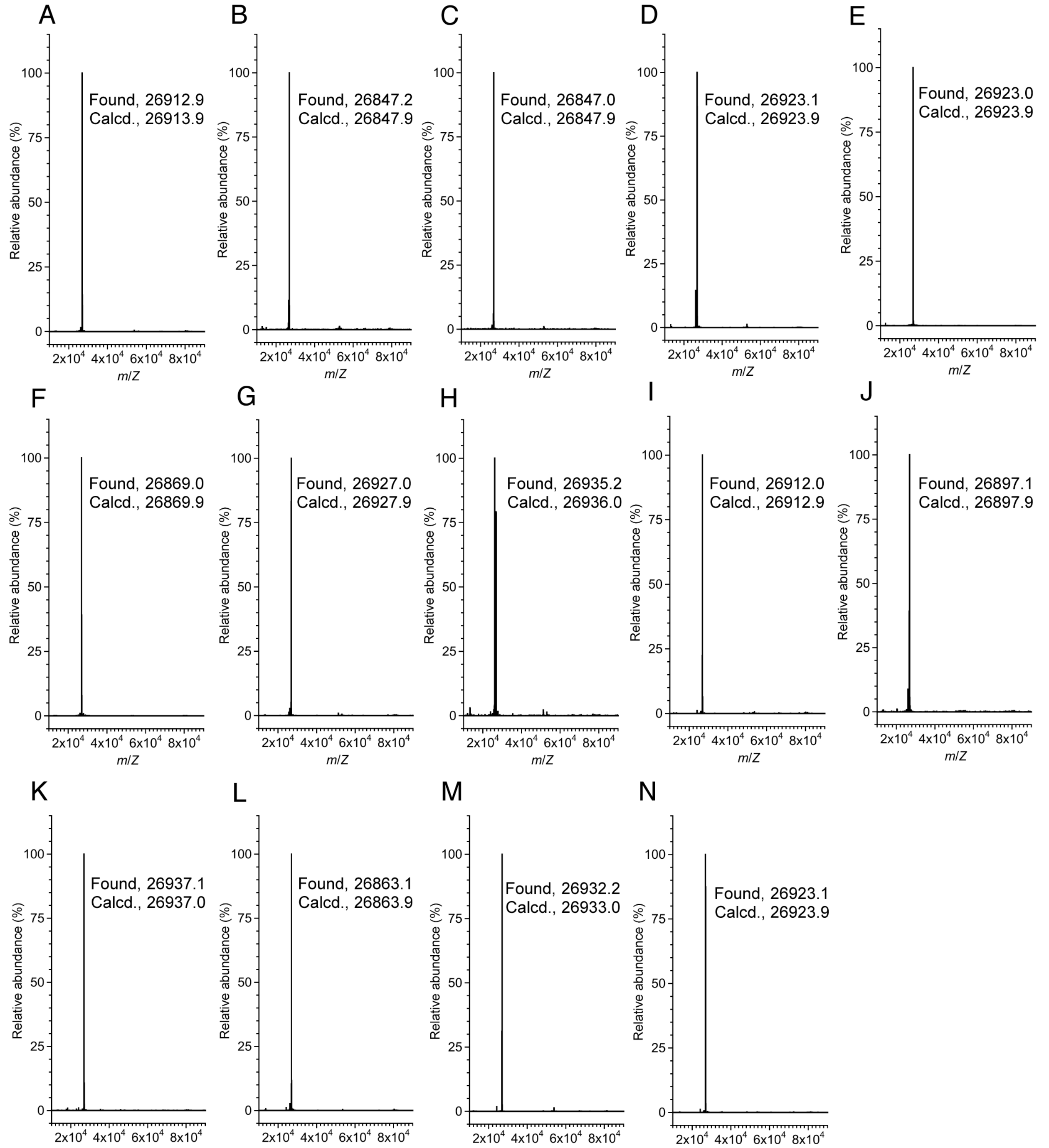

Fig. S11 Deconvoluted ESI-MS spectra of the 6-phosphogluconolactonase (**6-PGLac**) isoforms used in this study. A) **6-PGLac** (same data to Fig. S4F); B) H9A; C) H95A; D) H67F; E) H104F; F) D131A; G) D131E; H) D131H; I) D131N; J) Y69F; K) Y69W; L) Y69L; M) H9R; N) H95F. The determined masses are almost identical to the calculated masses derived from the corresponding amino acid sequence without the initial Met (Fig. S2).

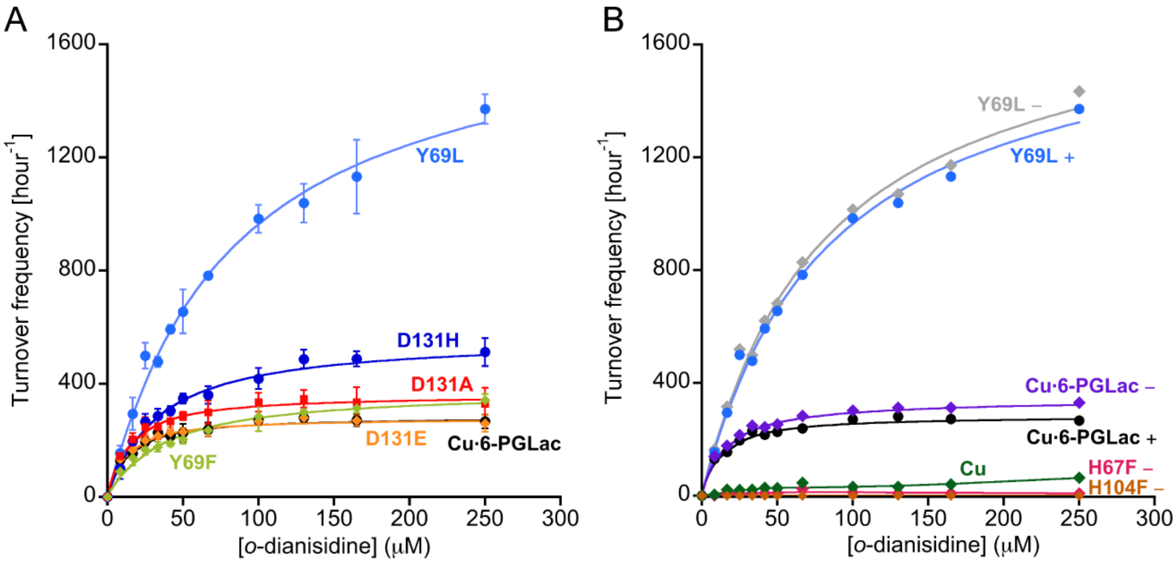

Fig. S12 Kinetic profiles of a nascent metalloperoxidase resulting from copper addition to 6-phosphogluconolactonase **6-PGLac**. (A); Michaelis-Menten saturation kinetics for the oxidation of *o*-dianisidine with *t*-BuOOH (6.6 mM) for selected mutants (1.25 μM) in the presence of copper (4.5 μM), [*o*-dianisidine]= 8-250 μM in MES-buffer (pH 6.5, 50 mM, 150 mM NaCl, 25°C). Measured data (symbols); fitted data (solid lines). The background reaction caused by free copper was subtracted from the raw data. Kinetic parameters are summarized in Table 1. (B) Comparison of the raw data (−) with the background-subtracted data (+). The background reaction in the presence of free copper reaction is highlighted in green.

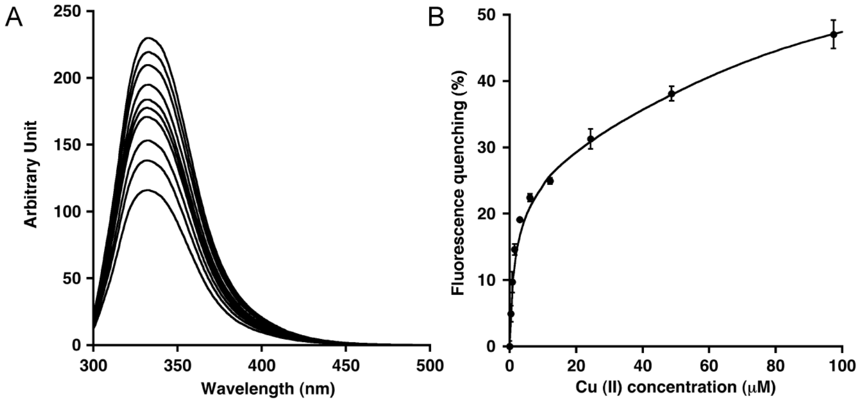

Fig. S13 Fluorescence spectral changes observed during the titration of **6-PGLac** (0.4 μM) with aliquots of $CuSO_4$ at 20 °C and pH 6.5 (50 mM MES and 150 mM NaCl). (A) 290 nm-excited fluorescence emission spectra of **6-PGLac** upon addition of $CuSO_4$ (0, 0.38, 0.76. 1.5, 3.0, 6.1, 12, 24, 49, and 97 μM, top to bottom). (B) Fluorescence quenching profile at 333 nm (in %) resulting from addition of $CuSO_4$ to **6-PGLac**. The fluorescence quenching intensity of **6-PGLac** exhibits a biphasic saturation curve, suggesting the presence of multiple Cu^{2+} binding sites. Analysis based on a two metal binding scheme provides a good fit with the following dissociation constants: K_{d1} = 0.83 ± 0.11 μM, K_{d2} = 130 ± 3.3 μM. The corresponding fluorescence quenching values (ΔF_1 and ΔF_2) are 22 ± 1.0 % and 57 ± 4.1 %, respectively.

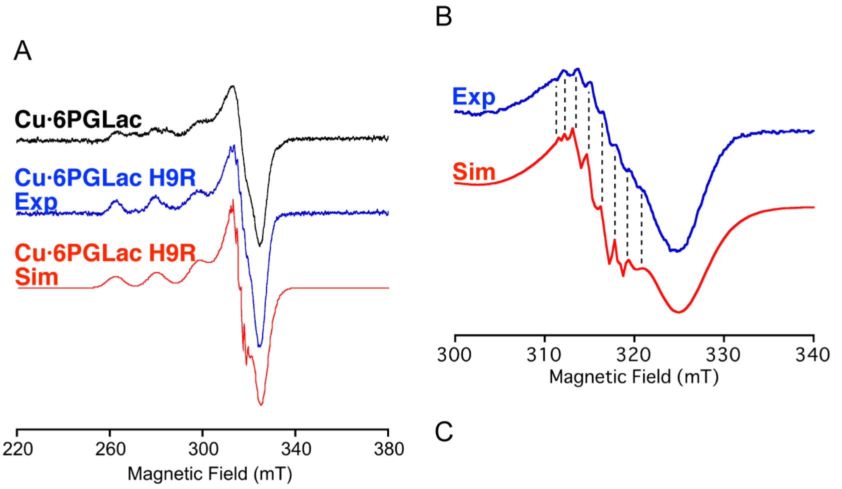

C

Cu

g_x = 2.038 A_x = 0.1 mT σ_x = 0.2 mT

g_y = 2.038 A_y = 0.1 mT σ_y = 10 mT

g_z = 2.230 A_z = 17.7 mT σ_z = 6.0 mT

N

Shfcc = 1.7 & 1.4 mT

Fig. S14 EPR spectra of **Cu·6-PGLac** and **Cu·6-PGLac** H9R with simulation at 77 K and pH 6.5 (50 mM MES and 150 mM NaCl). (A) Comparison among **Cu·6-PGLac** (black), **Cu·6-PGLac** H9R (blue), and **Cu·6-PGLac** H9R simulation (red). (B) Comparison between the measured (blue) and the simulated (red) region of H9R in the super hyperfine splitting of perpendicular region. (C) Detailed EPR parameters of **Cu·6-PGLac** H9R determined by EPR simulation. Although the intensity of superhyperfine splitting in the perpendicular region is insufficient for the accurate simulation, an overall fit could be achieved by introducing the parameters of two non-equivalent nitrogen atoms (1.4 mT and 1.7 mT, Fig. S14B and C). This is consistent with the Cu1 binding site observed in the crystal structure of **Cu·6-PGLac** with two non-equivalent Cu–N bonds.
